# Assessing the Need for Mental Health Support From Free-Text Responses: Development and Validation of Language-Based Assessments in Adults With Internalizing Symptoms

**DOI:** 10.2196/105460

**Published:** 2026-09-25

**Authors:** Clara Wiebel, Veerle C Eijsbroek, Vasudha Varadarajan, Katarina Kjell, H Andrew Schwartz, Oscar Kjell

**Affiliations:** 1 Department of Psychology, Lund University, Box 213, Allhelgona Kyrkogata 16A, 16B, 18A, 18B och 18C, Lund, Skåne, 22350, Sweden, +46 46 222 00 00; 2 School of Management, Technical University of Munich, Heilbronn, Baden-Württemberg, Germany; 3 Language Technologies Institute, Carnegie Mellon University, Pittsburgh, PA, United States; 4 College of Connected Computing, Vanderbilt University, Nashville, TN, United States

**Keywords:** mental health support, language-based assessments, natural language processing, machine learning, large language models, best-estimate assessments, AI

## Abstract

**Background:**

Machine learning and natural language processing have demonstrated significant potential for mental health assessment: describing your mental health in your own words can offer a more ecologically valid approach than traditional rating scales. However, most models focus on specific diagnoses, conditions, or symptoms, which may prematurely assign labels and potentially reinforce stigma in the context of early-stage mental health screening.

**Objective:**

This study develops a language-based assessment model that assesses the need for mental health support based on probed natural language and validates it against best-estimate assessments from multiple experienced psychotherapists.

**Methods:**

We analyzed an enriched online sample (n=600 for development and n=212 for validation), in which about half reported experiencing internalizing symptoms (depression or anxiety). Participants described their mental health using open-ended responses regarding (1) mental health, (2) suicidal thoughts, (3) medical history, and (4) depression. The responses were converted into contextual word embeddings using a large language model and entered as predictors in a ridge regression using nested cross-validation. Two to three experienced psychotherapists assessed each participant’s need for mental health support on a scale from 1 (no support needed) to 5 (potential crisis). Their assessments were based on longitudinal clinical data (natural language, validated scales, clinical interview, sociodemographics, and clinical history) and were averaged into a best-estimate assessment for model validation. We used the Sequential Evaluation With Model Preregistration framework, which separates model development from validation in a held-out set to support robust estimations and generalizability.

**Results:**

The language-based assessments closely aligned with the best-estimate assessments (*r*=0.82) and showed strong correlations with established clinical rating scales for depression (Patient Health Questionnaire-9), anxiety (Generalized Anxiety Disorder 7-Item Scale), stress (Perceived Stress Scale 10), and suicidality (Inventory of Depression and Anxiety Symptoms; *r*=0.62-0.77). Language-based visualizations of topics and word embeddings showed that low need for support assessments was associated with mentioning well-being and good health, while high assessments were related to depression, anxiety, and suicidality.

**Conclusions:**

This study demonstrates that natural language responses analyzed through large language models and machine learning can be used to assess individuals’ need for mental health support in close alignment with best-estimate assessments from experienced psychotherapists. Using less than 5 minutes of respondent time, this approach offers a practical tool for early-stage mental health screening in both clinical and self-guided screening contexts.

## Introduction

Nearly half of EU (European Union) residents reported emotional distress within the past year, such as feeling depressed or anxious [1]. Yet, even in high-income countries, only 23% of people with mental health issues receive minimally adequate treatment [2]. Personal barriers play a key role in this treatment gap: across 6 countries in North and South America, nearly 40% of people who did not seek any treatment for their mental health issues reported that they did not perceive a need for it [3]. Among those who recognized a need, the most common barriers were attitudinal, such as believing that their issues were not serious enough.

These patterns reflect what the literature terms unmet need for mental health care, which can arise at 3 stages on the pathway to care: not perceiving a need, not seeking care, and not receiving adequate care [4]. The first stage is pivotal, since perceiving a need strongly shapes whether people seek and use services [5]. Yet, perceived need is captured through self-report instruments such as the Perceived Need for Care Questionnaire [6] that depend on individuals recognizing a need themselves. This self-recognition is often absent precisely where clinical need is present: even among people who meet diagnostic criteria, those who do not perceive a need for care nonetheless show elevated distress and impairment [7]. There is thus a gap for accessible, early-stage assessments of support needs that do not depend on the individual already recognizing that need.

Someone’s need for mental health support can arise from a wide array of biological (eg, genes), developmental (eg, aging), psychological (eg, coping style), social (eg, loneliness), and structural (eg, discrimination) factors [8-10]. Rather than constituting need directly, these factors are determinants that shape the need for mental health support largely by contributing to psychological distress. In the current study, we assess the need for mental health support within the scope of internalizing symptoms (depression or anxiety) and suicidality. While internalizing symptoms do not encompass all possible manifestations of psychological distress (eg, externalizing symptoms), they represent one of the most prevalent, consistently used, and validated indicators of psychological distress in both research and clinical practice, while suicidality functions as a key marker of distress severity and urgency of need [11-13].

One promising approach for assessing need for mental health support is language-based assessments (LBAs), where an individual’s language use is analyzed to assess their mental state. The analysis of language use patterns—how people express their emotions, thoughts, or experiences in their own words—has provided valuable psychological insights for over multiple decades [14,15]. Recent advances in natural language processing and machine learning, particularly large language models (LLMs), have substantially improved the accuracy of LBAs [16,17]. Accordingly, a growing body of work has applied natural language processing and LLMs to assess and understand mental illness. Within the scope of internalizing symptoms and suicidality, studies have analyzed social media posts [18,19], text responses and essays [20,21], and text messages and conversations [22,23], as well as clinical interview and psychotherapy transcripts [24-26], collectively yielding meaningful insight into depression, anxiety, and suicidality symptoms, profiles, and trajectories. A related line of research has explored probed LBAs, where individuals describe their symptoms and related situations in response to targeted open-ended mental health questions [27]. Probed LBAs have been proposed as an ecologically valid complement to traditional assessment methods because they offer respondents more flexibility than closed-ended rating scales [28,29]. LLM-based analysis of probed responses has achieved convergent validity with rating scales approaching the scales’ own reliability—a theoretical upper limit of assessment accuracy [30,31].

Despite these advances, current LBAs are restricted in several ways in relation to assessing the need for mental health support. First, most models focus on specific diagnoses, conditions, or symptoms, which may disregard psychiatric comorbidities and prematurely assign diagnostic labels, potentially reinforcing stigma and discouraging help-seeking [32,33]. More recently, a smaller set of studies has moved toward broader dimensional or transdiagnostic framings [12], modeling spectra such as internalizing distress, rather than predicting diagnostic categories [34,35]. While these approaches can better capture comorbidity and reduce reliance on diagnostic labels, they remain oriented toward characterizing symptom dimensions or disorder spectra rather than directly assessing an individual’s need for support. While assessing internalizing symptoms and especially suicidality directly relates to the need for mental health support, we argue that assessing overall need first, rather than focusing on a specific condition or spectrum, can avoid these issues. It is potentially better suited for early-stage screening, where a formal diagnosis or condition-specific severity estimation is neither necessary nor appropriate. Although LBAs have successfully advanced the identification of internalizing symptoms and suicidality, to our knowledge, no validated instrument to date screens for the need for mental health support using natural language without relying on a specific diagnosis, condition, or spectrum. Meanwhile, broader screening tools such as the General Health Questionnaire [36] rely exclusively on rating scales.

Second, many LBAs are trained on passively collected language, such as social media posts [18], text messages [22], medical records [37], or interview or therapy transcripts [25]. While valuable, these sources are not universally accessible or applicable for early screening: not everyone maintains a social media profile or produces sufficient digital text for analysis; personal messages raise privacy concerns; medical records vary in availability and format across health care systems; and interview or therapy transcripts are only generated once someone has already moved past the point of early screening. Probed language responses, where individuals answer the same targeted questions, provide more standardized input that isolates the constructs of interest (and typically align the language source in time and content with the outcome, producing high convergent validity [28,30]).

Third, models are typically validated against a reference standard based on a single measure, such as rating scales [38] or structured interviews [39]. This limits their validity, since, in essence, every single measure of a psychological construct is prone to some sort of error or bias, such as recall bias in self-report scales [40] or interviewer bias in clinical interviews [41], and therefore they are unable to be a best estimate of a clinical construct [42,43].

Finally, even when machine learning models perform well on training data, they often fail to generalize to unseen data, which is a problem well-documented across predictive modeling in clinical science [44], suggesting that without rigorous validation procedures, such as testing preregistered models on held-out samples, reported accuracies may be overly optimistic.

This study addresses several limitations of previous approaches. First, it introduces assessments of the need for mental health support as an early complement or alternative to single-label diagnoses or severity estimations of specific conditions or symptoms. Need for support aims to account for comorbid mental illnesses and offers a less prescriptive approach. Conceptually, the assessments resemble the judgment of a mental health professional right after an initial consultation—on a clinician-rated scale ranging from (1) no support needed to (5) potential crisis. As the output is framed as supportive guidance rather than a diagnostic label or condition-specific severity estimation, it has the potential to be suited not only for clinical screening and monitoring, but also for guided self-screening contexts such as digital health platforms, where individuals can receive advice framed as supportive rather than clinical, indicating whether seeking professional support may be beneficial.

Second, our approach relies on structured, probed language responses rather than passively collected language. Respondents answer targeted mental health questions, either by writing brief paragraphs or selecting descriptive words from a predefined list [45]. This structured input produces standardized and comparable data, enhancing real-world applicability. It also enables examining whether a single general mental health question is sufficient to capture most needs, or whether additional questions, such as explicitly asking about medical history or suicidality, are needed to improve accuracy.

Third, the LBAs are validated against best-estimate assessments instead of a single measure. These best-estimates are obtained through a Longitudinal Expert All Data (LEAD) approach [46,47]: multiple experts assessed a case based on longitudinal clinical data (natural language, validated rating scales, clinical interview, sociodemographics, and clinical history), and their assessments are averaged to a “best-estimate” of the need for mental health support. Finally, to produce a fair estimation of replicability and generalizability, the current study adheres to the Sequential Evaluation With Model Preregistration (SEMP) framework [48]. In the development phase, the optimal combination of input variables that balances accuracy and parsimony is identified and preregistered. In the validation phase, preregistered models and hypotheses are tested in a separate held-out set, evaluating the LBAs model’s criterion, convergent, and face validity.

We hypothesize that the LBAs show strong criterion validity by correlating positively with best-estimate assessments from experienced psychotherapists as reference standard (H1). For convergent validity, we expect the LBAs to correlate positively with established measures of depression (H2a), anxiety (H2b), stress (H2c), and suicidality (H2d), and negatively with satisfaction with life (H2e) and harmony in life (H2f). For external validity, we expect positive correlations with self-reported sick days (H3a) and health care visits due to mental health (H3b) as behavioral indicators of mental health service use. Finally, we expect the LBAs to show face validity, with linguistic topics aligning with established psychological theories, specifically the broaden-and-build theory [49] regarding low need for support and the theory of depression [50,51] regarding high need for support (H4). The broaden-and-build theory [49] suggests that positive emotions expand cognitive and behavioral repertoires, facilitating well-being and social connectedness. Therefore, we expect LBAs of a low need for support to be associated with topics referring to, for example, social relationships, meaningful activities, recovery from stressors, a lack of mental or physical issues, and explicit affirmations of well-being. Conversely, the theory of depression by Beck [50] emphasizes that negative thought patterns and attentional biases toward distress lead to negative affect, which Clark and Watson [51] identify as a key feature of both depression and anxiety. Accordingly, we expect LBAs of a high need for support to be associated with topics reflecting negative thoughts, negative affect, and distressing life circumstances.

## Methods

### Procedure

The participants’ mental health data used to develop and validate the LBA model were drawn from a previously conducted longitudinal study focusing on assessing depression, anxiety, stress, suicidality, and self-harm [45]. Participants completed a comprehensive assessment survey at the beginning and end of the current study’s period, as well as shorter biweekly surveys in between. The surveys assessed mental and physical health through 3 response formats: brief written paragraphs (text responses), writing or selecting words from a list (word responses), and standardized rating scales. The order of the response formats (ie, open-ended questions or rating scales first) was randomized. The median estimated response time for the 4 language responses included in the final model (see the Model Development section) was approximately 4.79 (mean 6.14, SD 7.84) minutes.

### Open Science

In accordance with the SEMP framework, the first preregistration [52] outlines the model development, student assessment procedure, sample size, and exclusion criteria. After model development, a second preregistration [53] specified the final models and refined hypotheses tested in a held-out set. The final models are available in the LBA model library [54] and can be explored at our public demo page [55].

### Participants

Participants from the United States and United Kingdom were recruited via Prolific [56] and followed online for 10 weeks between June and December 2021. The participant sample was enriched, with approximately half of the participants self-reporting having been diagnosed with a mental health diagnosis of major depressive disorder (MDD [57]; ≈25%) or Generalized Anxiety Disorder (GAD [57]; ≈25%). Of the total sample, 212 participants were assigned to a held-out validation set based on having received assessments from multiple psychotherapists, and 600 were assigned to the development set (see the Selection of Development Sample section).

The sample was predominantly female (development: 396/600=66%; validation: 114/212=53.8%) with a mean age of 43.9 (SD 18.2) years in the development set and 40.2 (SD 13.0) years in the validation set. Participants in the validation set were, on average, slightly younger, more likely to hold a university degree, more likely employed, and more often male compared to the development set. Full sociodemographic details are presented in Figure 1 and Multimedia Appendix 1.

**Figure 1.**
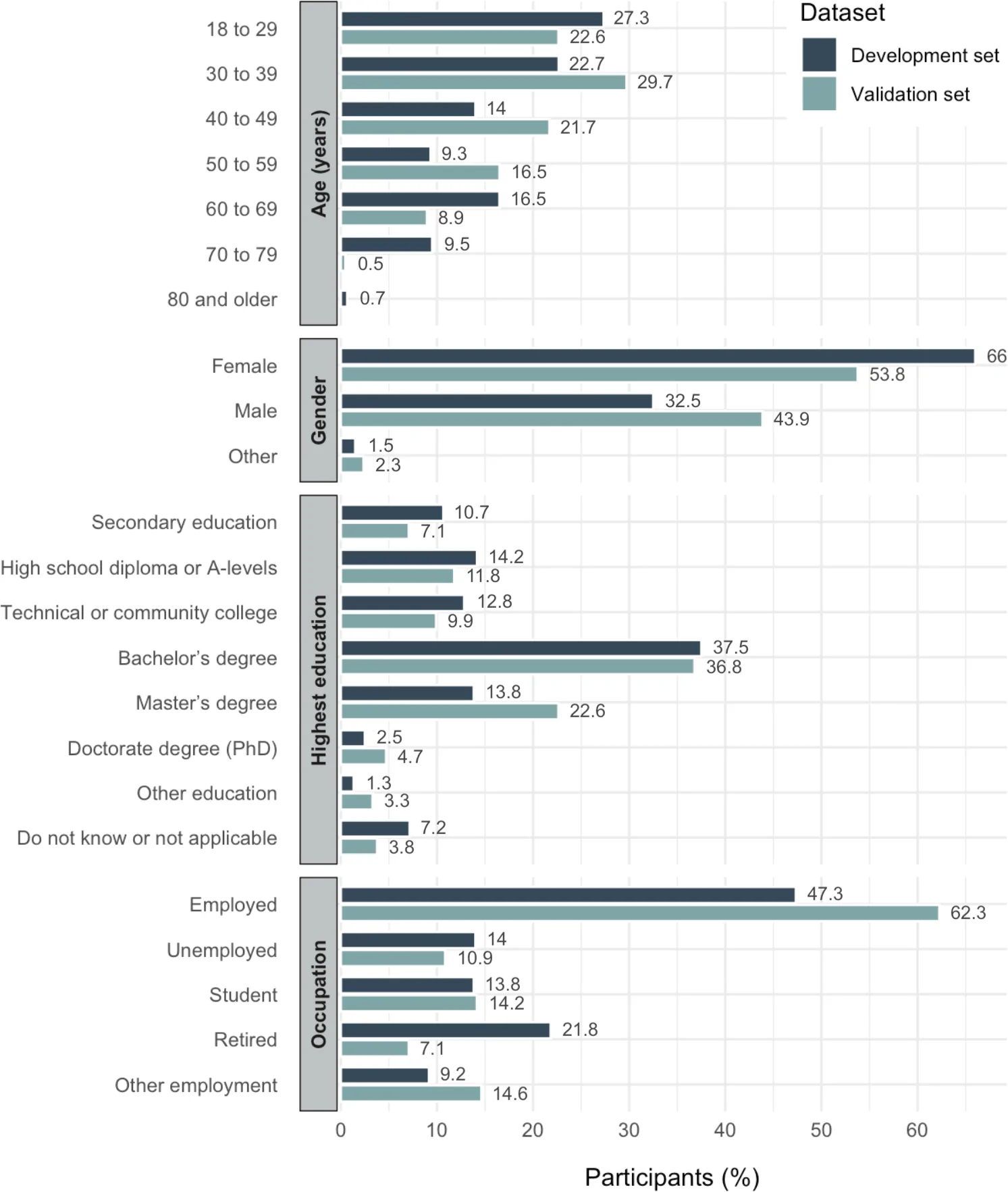
Sociodemographics in the development (n=600) and validation set (n=212). Participants could choose multiple options for the occupation variable.

### Measures

#### Natural Language Responses

Six text response questions and 2 word response questions were chosen from Kjell et al [45] based on clinical expertise being most relevant for determining need for mental health support and used as possible inputs for the LBA model. The six text response questions prompted participants to write brief paragraphs about their (1) mental health, (2) social situation, (3) support system, (4) medical history, (5) self-harm, and (6) suicidality. The two questions for word responses prompted participants to choose at least five words from a list of 20 words that describe their (1) mental health and (2) depressive symptoms over the past two weeks. Additionally, participants could also write their own descriptive words in an open text field. The exact wording of the questions included in the final model is presented below (see Table S1 in Multimedia Appendix 1 for all text and word response questions).

The mental health text response question asked:

*How is your mental health? Please describe how you have been over the last two weeks. You can, for example, write about your emotions, thoughts, behaviors, and/or symptoms related to your health. Write at least one paragraph.*

The suicidality text response question asked:

*Please describe whether, and if so how, you have been thinking about death, have had thoughts about killing yourself, have any intent or plan to kill yourself, or if you have attempted suicide over the last two weeks? If you have not had or done this, please briefly describe that in your own words.*

The medical history text response question asked:

*Describe your previous medical history; for example, when did you get sick, have you had similar or other problems before?*

The depression word response question asked:

*Write or select five words that best describe the level of depression that you have experienced, or not, over the last two weeks.*

*Please choose all that apply: content, motivated, optimistic, happy, hopeful, fearful, energetic, lazy, okay, upbeat, suicidal, anxiety, angry, alone, numb, pessimistic, empty, lethargic, dejected, despondent, other: (specify).*

#### Rating Scales and Behavioral Measures

The Patient Health Questionnaire-9 (PHQ-9) [58] is a 9-item instrument that measures the severity of depression. The items correspond to the diagnostic criteria for MDD [57]. Respondents rate how frequently they have experienced each symptom over the past 2 weeks on a scale of 0 (“not at all”) to 3 (“nearly every day”). An example item is “Over the last two weeks, how often have you been bothered by little interest or pleasure in doing things?”

The Generalized Anxiety Disorder 7-Item Scale (GAD-7) [59] measures the severity of GAD symptoms on a 7-item scale. Respondents indicate how often they have experienced symptoms of GAD over the past 2 weeks on a scale of 0 (“not at all”) to 3 (“nearly every day”). An example item is “Over the last two weeks, how often have you been bothered by feeling nervous, anxious, or on edge?”

The Perceived Stress Scale 10 (PSS-10) [60] is a 10-item instrument that assesses the frequency of subjectively experienced stress over the past month. Each item is answered on a scale of 0 (“never”) to 4 (“very often”), for instance, “In the last month, how often have you felt difficulties were piling up so high that you could not overcome them?”

The Inventory of Depression and Anxiety Symptoms (IDAS) [61] assesses 10 symptom dimensions of MDD and related anxiety disorders using 64 items. Participants rate the extent to which they experienced each symptom in the past 2 weeks on a scale of 1 (“not at all”) to 5 (“extremely”). This study exclusively uses the suicidality dimension, which consists of 6 items; for example, “I thought the world would be better off without me.”

The Satisfaction With Life Scale 3 (SWLS-3) and the Harmony in Life Scale 3 (HILS-3) [62] are abbreviated 3-item versions of their respective full scales, measuring global life satisfaction and harmony in life [63,64]. Both scales use a 7-point response format ranging from 1 (“strongly disagree”) to 7 (“strongly agree”). Example items include “In most ways, my life is close to my ideal” for the SWLS-3 and “Most aspects of my life are in balance” for the HILS-3.

The self-reported behavioral measures used in this study are the self-reported number of sick days and health care visits taken due to mental health issues over the past 3 months.

#### Need for Mental Health Support

The Need for Mental Health Support Scale was developed to assess the need for support based on multiple sources of longitudinal data [45]. It is completed by trained assessors (not self-reported)—in this study, psychotherapists and graduate students of psychology. The assessors could choose between 5 levels ranging from 1 (no support needed) to 5 (potential crisis; Table 1). The tool was designed with 2 audiences in mind: psychotherapists assessing the level of support needed, and those assessed who may receive the output. Notably, item formulations 4 and 5 start similarly to avoid alarming language, as these might be shown to those assessed; the distinction between the 2 levels is conveyed through the accompanying descriptions, which include more urgency and emergency information for level 5.

**Table 1.** Need for mental health support scale. The instruction to the assessors was “Which recommendation would you give the patient based on their overall responses?” The item formulations are addressed to those assessed because the resulting recommendation may later be shown to them.

Label	Item formulation
No support needed	1=Your mental health appears to be well. Your responses suggest that you do not need to consider seeking help for common mental health issues (ie, depression, anxiety, or stress).
Heightened attention	2=Consider paying attention to your mental health. Your responses suggest that although you do not have to seek help for common mental health issues (ie, depression, anxiety, or stress) at this point, there are things you can do on your own to improve your mental health. You can, for example, exercise, meditate, eat well, be more mindful, etc. If things are getting worse, consider reaching out to healthcare.
Consider seeking support	3=Consider talking to a mental health clinician. Talking to a mental health expert can help you increase your mental health before it gets worse. They can, for example, help you get the right help and give support.
Seek help (noncrisis)	4=Seek help from a mental health clinician. Talking to mental health clinicians can help you get the right support; this may, for example, include therapy and/or medication, which research has shown can help decrease common mental health problems. You may also get support from available helplines such as the Samaritans [65], 116 123 in the UK or the National Suicide Prevention Lifeline [66], 1-800-273-TALK (8255) in the US.
Potential crisis	5=Seek help from a mental health clinician. Getting help from mental health clinicians can help you in many ways. If you need to reach immediate care, please contact your local emergency number such as 999 or 112. You may also get support from available helplines such as the Samaritans [65], 116 123 in the UK or the National Suicide Prevention Lifeline [66], 1-800-273-TALK (8255) in the US.

### Assessment Procedure

#### Best-Estimate Assessments

Participants in the held-out validation set received a best-estimate assessment according to the LEAD method (described according to the LEADING reporting guideline [47] in Table S2 in Multimedia Appendix 1), which we used for model validation. As posed by Spitzer [46], the LEAD method establishes a near-ground truth (“best-estimate” [47]) for evaluating the validity of psychiatric assessments: participants are followed longitudinally and assessed by experts who have access to all collected data. Accordingly, experienced psychotherapists provided mental health assessments based on comprehensive, structured reports that included all information from the longitudinal surveys (eg, language responses, rating scales, sociodemographics, unstructured clinical interview, and clinical history; Figure 2 [28]). Each case was assessed by 2 or 3 licensed psychotherapists (≥5 y clinical experience; 2 women, 1 man; mean age 50.3, SD 12.9 y), and their assessments were averaged to form the best-estimate used as the reference standard.

**Figure 2.**
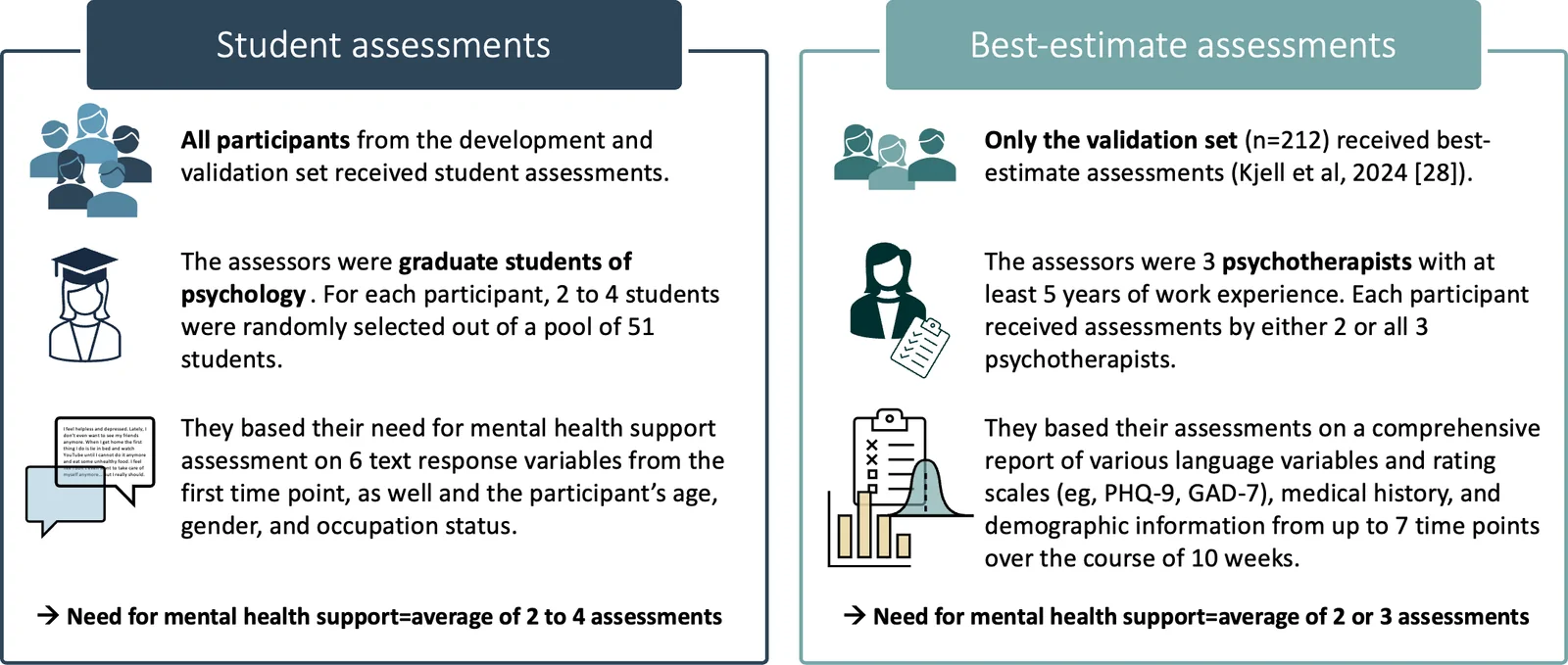
Overview of the student and best-estimate assessments for need of mental health support. For 167 cases in the development set, the student assessments were combined with a single psychotherapist assessment. GAD-7: Generalized Anxiety Disorder 7-Item Scale; PHQ-9: Patient Health Questionnaire 9-Item Scale.

#### Student Assessments

Participant responses in the development set (n=600) were assessed by graduate students of psychology (Figure 2). Each participant received 2 to 4 independent assessments, which were averaged for model training. Unlike the psychotherapists who reviewed all longitudinal data, the students based their assessments solely on the 6 text responses as well as the participant’s age, gender, and occupation status. Moreover, they only saw responses from the first assessment survey, not longitudinal data.

The student assessments were collected via an online survey on SoSci Survey [67] (Figure S1 in Multimedia Appendix 1). Each student assessed 40 participants, who were allocated semirandomly by the survey software so that each participant would receive an equal number of assessments. Four assessors (160 assessments) were excluded for completing the survey in under 15 (median completion time 45.03, IQR 35.57-80.60) minutes. The number of assessments per participant therefore varied between 2 and 4 (in the development set: 188, 31.3%, received two assessments; 392, 65.3%, three assessments; and 20, 3.3%, received four assessments).

The 51 final graduate student assessors were aged between 21 and 32 (mean 25.63, SD 2.62) years; 39 (76.5%) students were female, 11 (21.6%) students were male, and 1 student identified as other. A substantial majority (n=42 or 82.4%) had previous experience with patients with psychiatric disorders, either in an inpatient (n=29 or 56.9%) or outpatient (n=25 or 49%) setting. Moreover, 36 (70.6%) assessors reported previous experience with psychological diagnostics, either in clinical (n=31 or 60.8%) or nonclinical settings (n=8 or 15.7%).

To evaluate how similar the best-estimate and student assessments were, the validation set (n=212) was also assessed by the graduate students. We calculated three interrater reliability metrics in the validation set using Krippendorff α: reliability (1) among the psychotherapists, (2) among the students, and (3) between the best-estimate and averaged student assessments.

### Ethical Considerations

This study received ethical approval from the Swedish National Ethics Committee (Dnr 2021‐01820). Participants were informed about this study and that participation was voluntary. Consent was obtained before starting this study. Where participants answered open-ended questions on suicide and self-harm, they were required to tick a box indicating that they understood that the survey was anonymous and that no one would be able to contact them in relation to their responses, and they were provided information about where they could retrieve help (eg, suicide prevention helplines). They were compensated through money (£7.5/h; a currency exchange rate of GB £1=US $1.37 was applicable) on Prolific. The data collection of student assessments was approved by the Ethics Committee of the University of Mainz (2024-JGU-psychEK-022). Student assessors were compensated through course credit.

### Model Development

#### Selection of Development Sample

The development set was drawn from the 1095 participants not included in the held-out validation set. Of those, 167 participants who had received one psychotherapist’s assessment were included, and the remaining 433 were randomly selected, yielding a total of 600. This sample size was chosen to balance training accuracy with feasible annotation effort, based on Gu et al [29], who showed that LBA models trained with 500 achieved accuracy within *r* =±0.05 of models trained with 963.

#### Model Configuration

Selected language responses (see the Selection of Language Variables section) were transformed into word embeddings and used as predictors in a ridge regression to assess the need for mental health support. Specifically, the second-to-last layer of the transformer-based LLM mxbai-embed-large-v1 [68] was used to generate the embeddings (Figure 3). This is a document-tuned transformer model, a type that has demonstrated state-of-the-art performance on the Massive Text Embedding Benchmark [69] and has been shown to consistently outperform base transformer representations for person-level psychological assessment [70,71]. The model (Mixedbread AI [68]; ≈335 million parameters; 1024-dimensional embeddings) was used off the shelf without fine-tuning and run locally via the *text* package [72]. A completed GUIDE-LLM checklist for reporting LLM use [73] is provided in the supplementary material (Table S3 in Multimedia Appendix 1).

**Figure 3.**
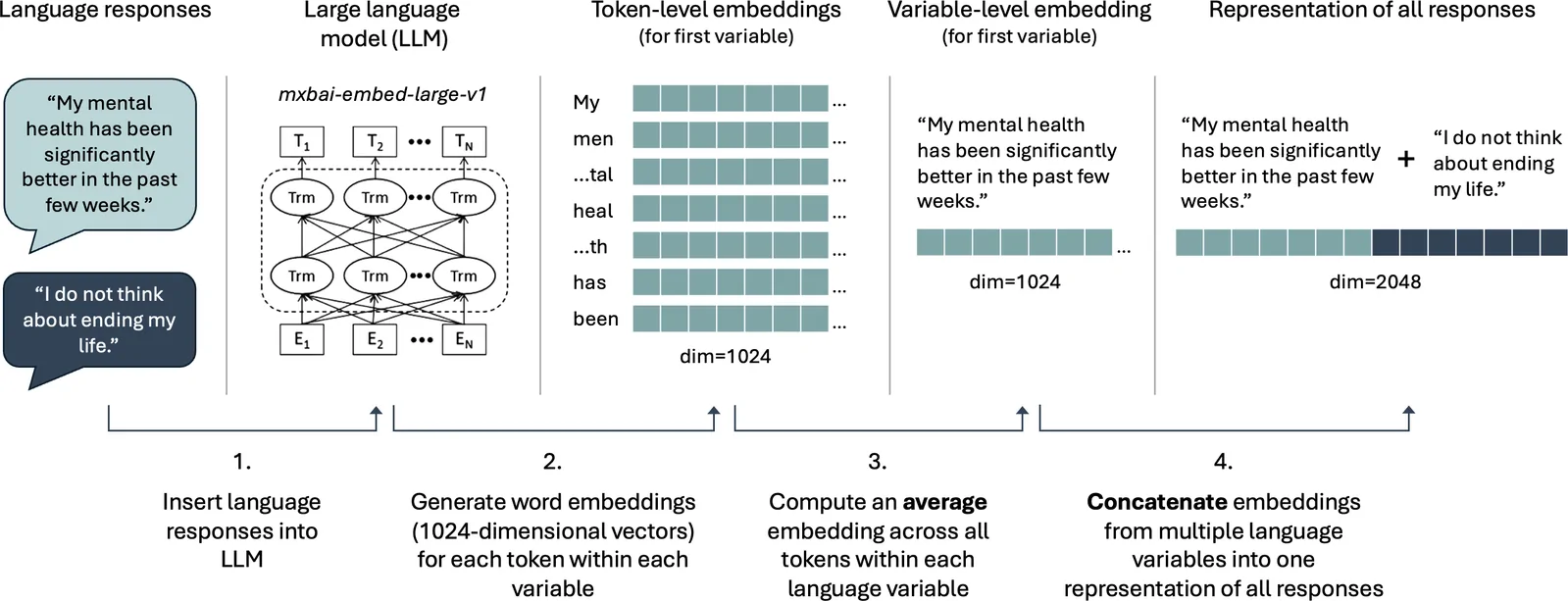
The embedding process for representing a participant’s language responses. Language responses are processed using the mxbai-embed-large-v1 model. Each response is tokenized, and a 1024-dimensional embedding is generated for each token. Token embeddings are then averaged across tokens to form a single representation for each language response. Finally, embeddings from different language responses are concatenated to create a unified representation for all responses. LLM: large language model.

The ridge regression was trained using 10-fold nested cross-validation to optimize the regularization parameter that minimizes overfitting. The search range was set from 10^-6^ to 10^6^. The model’s predictive accuracy was assessed using the Pearson correlation between actual and predicted values in the outer test folds. For some cases, the final LBAs were slightly below 1 or above 5, which were truncated to 1 and 5 for subsequent analyses.

During model development, we explored 3 methods to optimize word embeddings and improve model accuracy: averaging embeddings, Matryoshka embeddings [74], and prepending the question to participant responses before embedding extraction.

#### Selection of Language Variables

The selection of input variables followed an exploratory stepwise-forward approach. A ridge regression was fitted using only the embeddings of the mental health text responses since this question allowed respondents to broadly discuss diverse aspects of their mental health. Subsequently, each remaining variable was added individually to create multiple 2-variable LBA models. The LBA models were then repeatedly evaluated using data-driven and design-driven criteria until no further variables were selected. The primary criterion was data-driven: a variable was only considered if adding it significantly reduced model residuals, assessed via a 1-sided paired *t* test with α=.05. Although our first preregistration specified a Fisher z test as the data-driven criterion, we ultimately used the paired *t* test on model residuals due to its greater statistical power. Among variables reaching significance, design-driven considerations guided the final selection, including model parsimony, similarity to previously selected variables, and potential impact on the respondent’s time burden.

### Model Validation

Criterion validity (H1) was evaluated using the Pearson correlation between the LBAs and the best-estimate assessments in the validation set (n=212). A correlation of *r*≥0.70 was set as the threshold for adequate criterion validity [75]. We additionally computed a 1-sided disattenuated correlation [76]. Disattenuation estimates what the criterion validity correlation would be if the reference standard were measured without error—that is, if the psychotherapists agreed perfectly on the need for mental health support. The correction is 1-sided because only the reference standard, not the LBA, is corrected for unreliability. We used the interrater reliability among the psychotherapists (Krippendorff α) as the correction factor. As error-free measurement is unattainable in practice, the disattenuated correlation should be interpreted as an upper-bound estimate rather than a point estimate.

Convergent validity (H2) was evaluated using Pearson correlations between the LBAs and rating scales in the validation set (n=212). We expected significant, at least medium-sized, correlations of |*r*|>=0.30 [77]; specifically, positive correlations with depression (PHQ-9; H2a), anxiety (GAD-7; H2b), stress (PSS-10; H2c), and suicidality (IDAS; H2d); and negative correlations with life satisfaction (SWLS-3; H2e) and harmony in life (HILS-3; H2f). External validity (H3) was evaluated using Pearson correlations between the LBAs and the behavioral measures. We expected significant, at least small (*r*≥0.10) [77], positive correlations with the number of sick days (H3a) and the number of health care visits (H3b).

We also explored a parsimonious model using only the mental health text as a predictor and evaluated its criterion, convergent, and external validity (H1-H3). Such a model offers practical advantages, as open questions about mental health are commonly included in studies, interviews, and existing datasets, and requires less response time. The significance of all validity correlations (H1-H3) was 2-sided tested. Despite the directional hypotheses, we retained 2-sided tests as the more conservative choice.

Finally, face validity (H4) was assessed by examining how linguistic topics correlate with the LBAs. Unlike H1-H3, which evaluated model performance on held-out data, this analysis aimed to provide insights into the model’s decisions through language patterns. Topics were extracted by applying latent Dirichlet allocation (LDA [78]; see the Language-Based Visualizations section) on the complete dataset (n=812). LDA is an unsupervised method that is fully independent of the word embeddings, the ridge regression, and the human assessments. This justifies using the full sample, in addition to the fact that a larger sample yields more stable and interpretable topics. Yet, this analysis should be interpreted as descriptive face validity evidence rather than a test on unseen data. An a priori power analysis determined that with 812 participants, α=.05, and 90% power, correlations of |*r*|≥0.113 would be statistically significant [79]. As topic-outcome correlations typically remain below |*r*|=0.30, we expected at least 4 significant topics per text variable and 2 significant topics per word variable.

### Language-Based Visualizations

We examined linguistic patterns associated with the LBAs using 2 complementary methods [80]: LDA [78] and supervised embedding projections [72]. LDA is a topic modeling method that identifies latent topics within a text corpus based on word frequencies and co-occurrences. We extracted 12 topics per text variable and 2 topics per word variable. Before topic extraction, stop words [81], repeated phrases from the questions, and highly frequent terms were removed, with frequency thresholds set individually per variable (ranging from 150 to 450 occurrences). Co-occurring words were structured into a Document Term Matrix, allowing for 1- to 3-grams. Topic distributions within each response were then used to predict the LBAs via linear regression, with standardized regression weights serving as effect sizes. False Discovery Rate corrections [82] were applied within each language variable.

Supervised embedding projections [72] provided a second, complementary visualization based on word embeddings rather than word frequency. This method identifies words significantly associated with the LBAs by comparing responses from the highest and lowest quartiles of the assessed need for support. Embeddings from both groups were aggregated, and a difference vector was computed. Each word was projected onto this vector using the dot product, mapping its association with the LBAs. Statistical significance was assessed against a permuted null distribution, with False Discovery Rate corrections [82] applied within each variable. Stop words [81] were removed before analysis.

### Statistical Software

All analyses were performed in R (version 4.4.1) [83]. The model development and supervised embedding projections were performed using the *text* package (version 1.2.3) [72]. LDA topics were extracted with the *topics* package (version 0.40.1) [84]. Data wrangling, descriptive analyses, and visualizations were carried out with the packages *dplyr* (version 1.1.4) [85] and *ggplot* (version 3.5.1) [86]. Krippendorff α was calculated using the *irr* package (version 0.84.1) [87].

## Results

### Descriptive Statistics

Participants wrote between a median of 9 with IQR 5-15 (self-harm) and 44 with IQR 30-68 words (mental health) in the text response variables (Table S4 in Multimedia Appendix 1). A substantial proportion of participants (development: 240/600 or 40%; validation: 94/212 or 44.3%) fell within clinically relevant ranges of at least moderate depression and/or anxiety according to PHQ-9 and GAD-7 thresholds [58,59]. All rating scales demonstrated good to excellent internal consistency in both the development and validation sets (Cronbach α=0.85-0.96; see Table S5 in Multimedia Appendix 1 for scale-specific values).

On average, participants’ need for mental health support in the validation set was 2.55 (SD 1.07) as assessed by the students and 2.50 (SD 1.16) as assessed by the psychotherapists; this difference was not significant, *t*_211_=1.064, *P*=.29. An exploratory analysis of where the student and best-estimate assessments converged and diverged is reported in Multimedia Appendix 1, as well as full descriptive statistics of the language responses, rating scales, and need for mental health support assessments (Tables S4 to S7 in Multimedia Appendix 1).

### Model Development

Comparing embedding optimization methods revealed that prepending questions to responses before embedding extraction significantly improved model performance (see Table S8 in Multimedia Appendix 1). This finding is theoretically plausible because contextual embeddings represent each token conditional on its surrounding text. Short responses in particular (especially in the suicidality text responses, eg, “no, never”) are often ambiguous in isolation, and prepending the corresponding question anchors the response to the construct being probed (eg, denial of suicidal ideation). Accordingly, this approach was adopted in all subsequent analyses.

Using only the mental health text response, the model achieved a training accuracy of *r*=0.79 (Table 2). In the first step of the step-forward procedure, adding suicidality yielded the largest increase in accuracy (*r*=0.79 to *r*=0.84). In the second step, medical history further improved accuracy (*r*=0.86). In the third step, only depression words significantly improved the model. Despite a small increase (Δ*r*=0.01), we included it because participants typically select these words in under a minute. The final 4-variable model achieved a training accuracy of *r*=0.87.

**Table 2.** Comparison of the selected models in the Stepwise-Forward approach (n=600). This table compares each model to the model in the row directly above through a paired, 1-sided *t* test of model residuals. See Table S9 (Multimedia Appendix 1) for all tested models.

Input variables	*r* ^a^	95% CI	Mean abs residual^b^ (SD)	∆ res^c^	*t* test (*df*)	*P* value
Mental health	0.79	0.76-0.82	0.55 (0.42)	N/A^d^	N/A	N/A
Mental health + suicidality	0.84	0.82-0.86	0.49 (0.36)	0.06	5.02 (599)	<.001
Mental health + suicidality + medical history	0.86	0.83-0.87	0.45 (0.36)	0.03	3.84 (599)	<.001
Mental health + suicidality + medical history + depression words	0.87	0.84-0.88	0.44 (0.35)	0.01	1.66 (599)	.049

a *r*: correlation between the actual and predicted values.

b Mean abs residual: mean absolute residual of actual and predicted values.

c res: mean difference of absolute model residuals.

d N/A: not applicable.

### Model Validation

The final model achieved a correlation of *r*=0.82 with the best-estimate assessments, 95% CI 0.77-0.86 in the held-out set, exceeding the *r*=0.70 threshold for criterion validity (H1; Table 3). Notably, the 2 individual psychotherapists agreed with each other at *r*=0.78, meaning the model’s alignment with the best-estimate (*r*=0.82) already exceeds the level of agreement between individual clinicians. Correcting for imperfect interrater reliability among the psychotherapists increased the correlation to nearly perfect (*r*_corrected_=0.96 with Krippendorff *α*_psychotherapists_ =0.73). This upper-bound estimate indicates that the observed *r*=0.82 is close to the maximum achievable given the reliability of the reference standard. Further, the correlation between the LBAs and student assessments was the same in the validation set as in the development set (*r*=0.87), indicating excellent generalization. The LBAs correlated more strongly with student assessments (*r*=0.87) than the best-estimate assessments did (*r*=0.82), likely because both the model and the students based their assessments on the same language responses.

**Table 3.** Criterion validity: correlations with best-estimate assessments in validation set (n=212). The comprehensive LBA^a^ model is trained on the mental health text, suicidality text, medical history text, and depression word variables; the parsimonious LBA model is trained solely on the mental health text. The third psychotherapist only assessed 101 participants and was therefore excluded from this table.

	1	2	3	4	5	6
Comprehensive LBA	—^b^	.86^c^	.82^c^	.77^c^	.73^c^	.87^c^
Parsimonious LBA	.86^c^	—	.77^c^	.72^c^	.68^c^	.76^c^
Best-estimate assessment	.82^c^	.77^c^	—	.94^c^	.93^c^	.82^c^
Psychotherapist 1	.77^c^	.72^c^	.94^c^	—	.78^c^	.77^c^
Psychotherapist 2	.73^c^	.68^c^	.93^c^	.78^c^	—	.72^c^
Average student assessment	.87^c^	.76^c^	.82^c^	.77^c^	.72^c^	—

a LBA: language-based assessment.

b Not available.

c *P*<.001 (2-sided test).

The LBAs further demonstrated strong convergent validity (H2; Table 4), including strong positive correlations with depression (*r*_PHQ_=0.77), anxiety (*r*_GAD_=0.72), stress (*r*_PSS_=0.76), and suicidality scores (*r*_IDAS_=0.62), as well as strong negative correlations with life satisfaction (*r*_SWLS_=−0.66) and harmony in life (*r*_HILS_=−0.64). All correlations were statistically significant (*P*<.001) and classified as large effects [77]. What is important to note is that the criterion and convergent validity estimates are not completely independent signals due to shared method variance: the rating scales were part of the data evaluated by the psychotherapists to achieve the best-estimate assessments, an inherent property of LEAD designs (ie, all data [46]; see the Discussion section). Regarding external validity (H3), the LBAs correlated significantly with the number of health care visits (*r*=0.16, *P*<.05), meeting the threshold for a small effect size [77], but not with the number of sick days (*r*=0.09, not significant). Distributions of the LBAs and human assessments, as well as analyses of model residuals, are reported in Figures S2-S5 in Multimedia Appendix 1.

The parsimonious model using only mental health texts demonstrated strong performance, only modestly lower than the full model. The LBAs showed a correlation of *r*=0.79 with student assessments during model development (Table S10 in Multimedia Appendix 1), and a correlation of *r*=0.77 with the best-estimate assessments in the validation set (Table 3). It showed significant large correlations with the PHQ-9, GAD-7, PSS-10, SWLS-3, and HILS-3, but a comparatively small correlation with IDAS suicidality scores (Table 4 and Table S11 in Multimedia Appendix 1). Correlations with the behavioral measures were in the expected direction, but did not reach significance.

In an exploratory post hoc analysis, we compared the LBAs against demographic, dictionary- and frequency-based baseline models trained and validated in the same pipeline. The LBAs outperformed all baselines, with the largest advantage for the parsimonious model in comparison to the language baseline models (see Figure S6 and Table S12 in Multimedia Appendix 1). In a further exploratory analysis, the baseline LBAs predicted depression, anxiety, stress, and suicidality scores 10 weeks later, beyond the respective baseline scale score (Table S13 in Multimedia Appendix 1)

**Table 4.** Convergent and external validity: correlations in validation set (n=212). The comprehensive LBA^a^ model is trained on the mental health text, suicidality text, medical history text, and depression word variables; the parsimonious LBA model is trained solely on the mental health text. The *P* values of the correlations refer to a 2-sided test.

Variable	LBA			
	Comprehensive		Parsimonious	
	Pearson *r*	*P* value	Pearson *r*	*P* value
Convergent validity				
PHQ-9^b^	0.77	<.001	0.74^c^	<.001
GAD-7^d^	0.72	<.001	0.72^c^	<.001
PSS-10^e^	0.76	<.001	0.75^c^	<.001
IDAS suicidality^f^	0.62	<.001	0.46^c^	<.001
Inverse convergent validity				
SWLS-3^g^	−0.66	<.001	−0.63^c^	<.001
HILS-3^h^	−0.64	<.001	−0.64^c^	<.001
External validity				
Sick days^i^	0.09	.21	0.06	.42
Health care visits^j^	0.16^c^	.02	0.11	.11

a LBA: language-based assessment.

b PHQ-9: Patient Health Questionnaire 9-item scale.

c Update footnote.

d GAD-7: Generalized Anxiety Disorder 7-item scale.

e PSS-10: Perceived Stress Scale 10-item version.

f IDAS suicidality: suicidality dimension of the Inventory of Depression and Anxiety Symptoms.

g SWLS-3: Satisfaction With Life Scale 3-item version.

h HILS-3: Harmony in Life Scale 3-item version.

i Sick days: number of sick days taken in the last 3 months due to mental health issues.

j Health care visits: number of health care visits taken in the last 3 months due to mental health issues.

Finally, the extracted LDA topics derived from the complete dataset (n=812) were well-interpretable for all 4 language responses (see Figure 4). We extracted 12 topics per text response variable and 2 topics per word response variable (see the Methods section). Eleven topics for mental health, 11 topics for suicidality, and 7 topics for medical history correlated significantly with the comprehensive LBA model. The significant topics ranged from |β|=0.08 to |β|=0.36. For the depression words, both topics were significantly associated with the LBAs, showing large associations of |β|=0.76. Overall, the number of significant topics exceeded the predefined threshold and aligned with established psychological theories [49-51], supporting face validity (H4).

**Figure 4.**
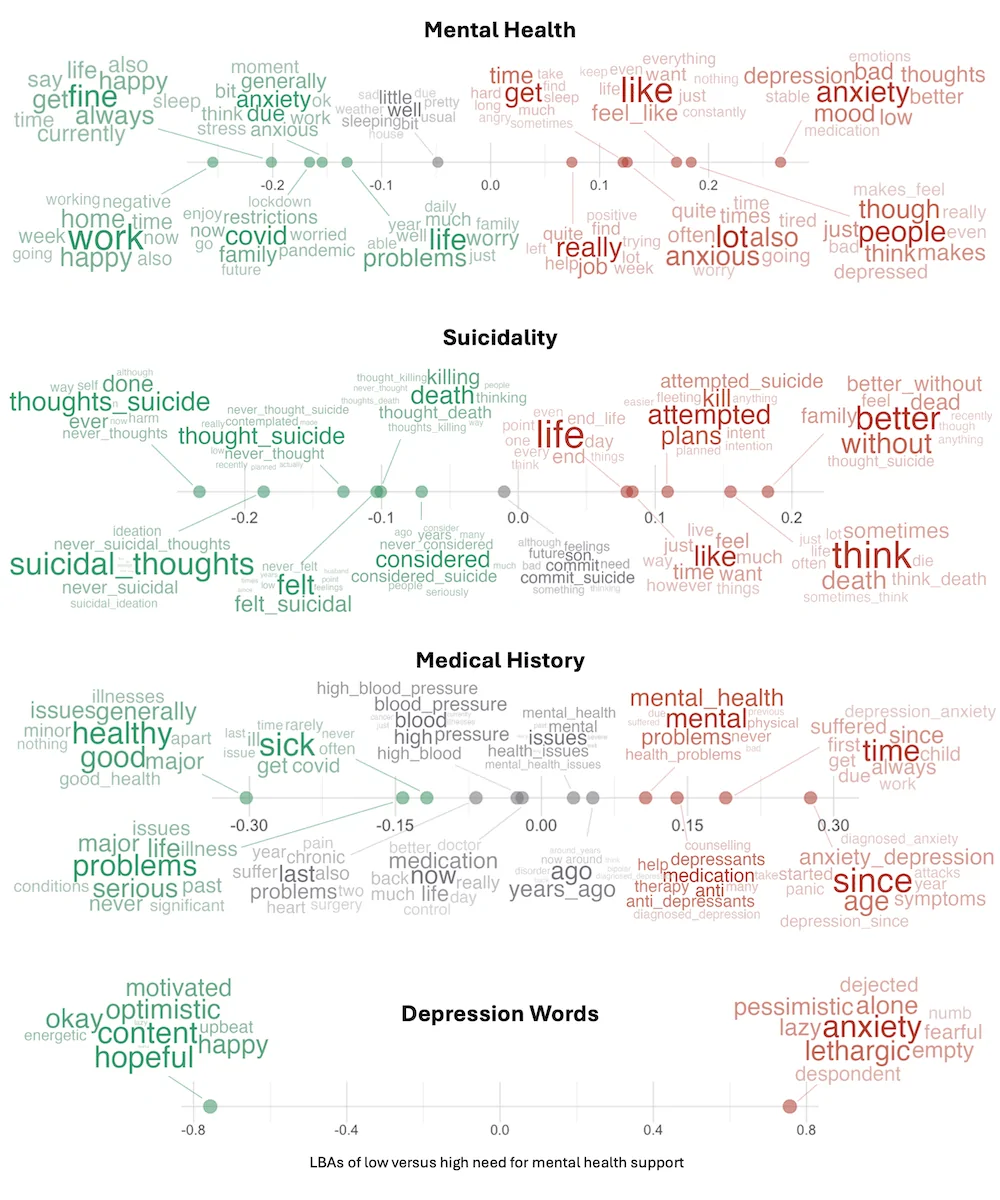
Topics associated with LBAs of high vs low need for mental health support (n=812). The horizontal scale shows the magnitude of the topic standardized regression weights (β), indicating the strength of the relationship between latent Dirichlet allocation (LDA) topics and the LBAs. The font color indicates their direction and significance: significantly negative (green), nonsignificant (gray), and significantly positive (red). Font size and transparency indicate the probability of a word within its topic. Topics were extracted and tested on the complete dataset (n=812), including the held-out validation set (see Methods for details). In the validation set, the topics‘ association with the LBAs closely mirrored their associations with the best-estimate assessments (Figure S7 in Multimedia Appendix 1). LBA: language-based assessment.

Topics significantly associated with LBAs of low need for support included “work,” “home,” “happy,” “fine,” the absence of suicidal thoughts, good health, and rarely getting sick. Prominent words in the associated depression words topic included “hopeful,” “content,” and “optimistic.” These topics align with the broaden-and-build theory [49] that suggests that positive emotions expand and facilitate cognitive and behavioral patterns, which increases well-being and social connectedness. Surprisingly, the mental health topics referring to anxiety, problems in life, and the COVID-19 restrictions were also significantly associated with lower need for mental health support, despite not being inherently positive.

Topics significantly associated with LBAs of high need for support referred to anxiety, depression, the perception of others (“people”); comparisons (“like”); thinking about, planning, or attempting suicide; and thinking that others would be better off without them. Within the medical history variable, both acute mental health issues (eg, taking psychiatric medication) and long-term mental health issues (indicated by words such as “since,” “age,” “time,” and “suffered”) were significantly related to higher LBAs. Prominent words in the associated depression words topic included “anxiety,” “lethargic,” and “pessimistic.” These topics correspond to theories of depression [50,51] that suggest that negative cognitive patterns and attentional biases lead to negative affect, which can lead to or reinforce symptoms of depression and anxiety.

The exploratory supervised embedding projections (in contrast to the LDA topics, only based on the held-out set; Figure 5) revealed many words significantly associated with LBAs of low or high need for mental health support. Overall, the direction and significance of words matched those found in the LDA topics (Figure 4). For instance, words such as “happy,” “good,” and “hopeful” were significantly associated with low need for support, while “anxiety,” “depression,” and “overwhelmed” were associated with high need. In the suicidality variable, words explicitly denying “suicidal” “thoughts” (eg, “not,” “never,” and “no”) were strongly associated with low need, whereas words describing suicidal intent (eg, “plans,” “attempting,” and “committing”) were associated with high need. Notably, the word “blood” in the projection plot of the medical history variable likely refers to high blood pressure, as inferred from the LDA topics. This example shows how LDA topics and 3-grams can provide slightly more contextual grouping, while supervised embedding projections more directly reflect the LBA model’s underlying process. Overall, these results further support the face validity of the LBAs by corroborating the LDA topic findings using a different method [80].

**Figure 5.**
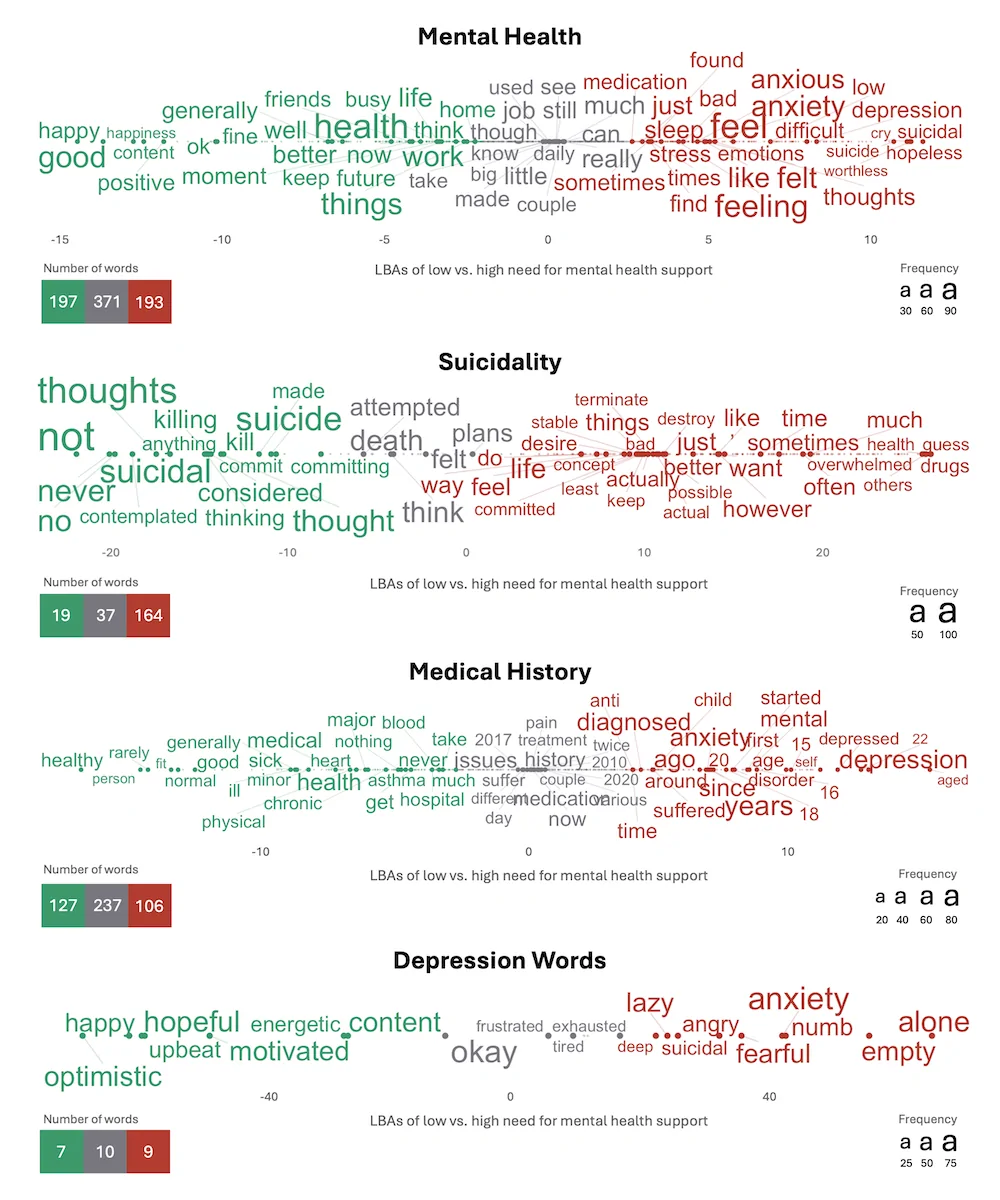
Words associated with LBAs of high vs low assessed need for mental health support (n=212). Each word is projected onto the semantic axis reflecting LBAs of low (left) vs high (right) need for mental health support. The boxes indicate the number of words significantly negatively correlated (green), not significantly correlated (gray), and significantly positively correlated (red) with the LBAs. The plots were created using supervised embedding projections [72]. LBA: language-based assessment.

## Discussion

### Principal Findings

This study developed and validated LBAs of need for mental health support using natural language responses. We validated 2 models: a parsimonious model using only a mental health text response, and a comprehensive model that additionally includes texts about suicidal thoughts and medical history, as well as depression-related words. Responding to all 4 language variables took participants less than 5 minutes in total.

Using the SEMP framework, models and hypotheses were preregistered before testing on a held-out validation set. The LBAs in the held-out set demonstrated strong criterion validity, correlating strongly with the best-estimate assessments of experienced psychotherapists who had access to longitudinal patient reports. The LBAs further showed strong convergent validity, correlating strongly with established measures of depression, anxiety, stress, and suicidality, and strongly negatively with established measures of well-being. Significant associations with linguistic topics were consistent with established psychological theories: low need for support assessments were associated with positive emotions, relationships, good health, and lack of suicidal thoughts, while high need for support assessments were linked to depression, anxiety, suicidal thoughts, and an ongoing history of mental illness. These associations were confirmed through 2 independent methods, LDA topic modeling and supervised embedding projections.

### Best-Estimate Reference Assessments

Much progress has been made in LLMs’ contextual language understanding and predictive accuracy [16,28]. However, for LLM-driven mental health assessments, the absence of a ground truth makes model training particularly complex. Best-estimate assessments are crucial for establishing a valid reference standard against which models can be evaluated, and averaging the assessments of multiple psychotherapists based on longitudinal clinical data represents one of the strongest available approaches [47,88]. It is important to note that the longitudinal clinical data evaluated by the psychotherapists also included the rating scales (ie, all data in the LEAD design [46]), which makes the benchmarks for criterion and convergent validity not completely independent due to shared method variance.

The observed interrater reliability among the individual psychotherapists (Krippendorff α=0.73) highlights that even experienced experts sometimes disagree on mental health assessments. Given that this reliability sets a theoretical upper limit on expected validity [30], it is notable that the LBAs achieved a correlation of *r*=0.82 with the clinicians’ best-estimate assessments. This suggests that the model captures a more stable signal than a single clinician, likely because it applies the same weighting of linguistic features consistently across cases, whereas individual clinicians may vary in what information they prioritize. However, the model lacks the clinical flexibility to recognize atypical presentations or contextual factors not captured in the text, which remains an important advantage of human judgment. This underscores the potential for a complementary approach, where the model provides a consistent baseline assessment that clinicians can then refine based on their expertise.

Remarkably, the assessments of the graduate students—on which the model was trained—converged closely with the best-estimate assessments, despite the students’ more limited clinical experience and their access to only the baseline language responses and demographics. Yet, an exploratory post hoc analysis indicated that the psychotherapists’ divergence from the student assessments was largely systematic rather than random. The difference between student and best-estimate assessments was substantially explained by the clinical rating scales that only the psychotherapists had seen (see Figure S8 and Table S14 in Multimedia Appendix 1 for the full variance-reconstruction analysis). Still, the high overall convergence supports training LBA models on assessments from carefully instructed raters when expert capacity is limited, provided that the validation is conducted against an expert-based reference standard.

### Incremental Value of Multiple Probed Language Responses

While the parsimonious model already demonstrated a high accuracy, adding an explicit question about suicidality significantly improved model performance. The parsimonious model seemed to particularly miss information about suicidality, as evidenced by the correlation with IDAS suicidality substantially increasing from the parsimonious to the comprehensive model. This advantage could also be observed for the criterion validity, yet it is partly attributable to how the criterion itself was constructed: the best-estimate assessments were based on psychotherapists’ evaluations of longitudinal clinical data centering on internalizing disorders, self-harm, and suicidality. Moreover, the psychotherapists assessed participants’ need for mental health support immediately after assessing self-harm and suicidality [45] (see Table S2 in Multimedia Appendix 1), likely inflating the correlation between these assessments. Besides design characteristics, suicidal ideation and especially intention are evidently directly related to someone’s need for mental health support. Suicidal ideation remains a highly stigmatized topic and is often not disclosed spontaneously [89], highlighting the importance of explicitly prompting for suicidality to enhance the sensitivity of LBA models to this critical issue. Crucially, meta-analyses and reviews show that actively asking individuals about suicide does not appear to induce or increase suicidal ideation [90,91], affirming that explicit screening is safe.

### Comparison With Traditional Language Analysis Approaches

To situate the contribution of the embedding-based approach within more traditional language analysis methods, we compared the LBAs with 2 established alternatives trained in the same pipeline: a tf-idf (term frequency-inverse document frequency) bag-of-words model and a model trained on a negation-aware suicide dictionary and LIWC-22 (Linguistic Inquiry and Word Count) dimensions [92]. The LBAs outperformed both baselines in the held-out set, while both language-based baseline models clearly outperformed the demographics-only model (see Figure S6 and Table S12 in Multimedia Appendix 1 for the full analysis). Notably, the advantage of the embeddings was modest when all 4 language variables were available, but substantial for the parsimonious models based only on the single mental health response. This shows that contextual embeddings maintain accuracy with brief responses, the scenario most relevant for large-scale screening.

Beyond these traditional baselines, our approach can be situated relative to generative encoder-decoder architectures increasingly used for LBAs by prompting an LLM to directly output a score from text. Zero-shot LLMs can approach human-rater accuracy but remain prompt-sensitive, performing best combined with document-tuned embedding-and-regression pipelines [93]. We do not argue for one method over the other, but a document-tuned encoder paired with ridge regression is well suited to person-level psychological assessment: its regularization supports stable estimates under sample size constraints, the model weights yield deterministic outputs that remain stable over time, and document-tuned LLM embeddings have been repeatedly benchmarked as strong, efficient representations of psychological constructs from text [94]. Practically, this architecture is also lightweight: with 335 million parameters, the model can be run on standard hardware and entirely on-site. In applied settings such as digital health care providers, sensitive free-text responses can be processed without transmitting them to external providers, at low computational cost.

### Limitations and Future Research

The LBAs correlated strongly with mental health scales, yet only partly with behavioral measures. The null finding for sick days may reflect the measure’s limited variance in this sample (n=190 or 90% of validation-set participants reported 0 sick days over the past 3 months), rather than an insensitivity of the LBA to functional impairment.

Comparing the LBAs to rating scales requires caution, since the best-estimate assessments were based on both language responses and rating scale scores, making them difficult to use as an independent benchmark. The students based their assessments solely on language responses, so it is unsurprising that the LBAs correlated more strongly with student assessments (*r*=0.87) than the PHQ-9 did (*r*=0.75). Conversely, the psychotherapists had access to rating scales alongside language and clinical data, which likely explains why the best-estimate assessments correlated slightly more strongly with the PHQ-9 (*r*=0.84) than with the LBAs (*r*=0.82). These patterns reflect what information each assessor had access to, rather than the inherent superiority of either method. However, a recent investigation of the PHQ-9 showed that most respondents misinterpreted its instructions, basing responses on perceived bothersomeness rather than symptom frequency as intended [95]—a limitation potentially shared by similar scales. While this points to the scales being imperfect benchmarks for convergent validity, it reinforces the value of the criterion validity analysis against the best-estimate assessments as the primary validation approach. Beyond accuracy, LBAs offer unique advantages: they capture nuanced, real-life expressions of mental health and avoid the rigidity of rating scales [16,28]. Additionally, clinicians can review the written responses themselves alongside the numerical score, enabling a hybrid approach in which statistical evaluation and clinical judgment complement each other.

The model exhibited a regression-to-the-mean effect by systematically overestimating low need for support cases and underestimating high need for support cases (see Figures S2-S5 in Multimedia Appendix 1). This is common in regularized predictive models since they penalize coefficients to prevent overfitting, which shrinks predictions toward the mean of the criterion [48]. This can be mitigated by distribution-based corrections, which realign the distribution of the predictions with that of the human assessments—either by transforming the criterion before training [48] or by remapping the predictions post hoc. Our demonstration (Multimedia Appendix 1) shows that post hoc transformations substantially reduce the overestimation of low-severity cases and underestimation of high-severity cases—without lowering criterion validity (Figure S9 and Table S15 in Multimedia Appendix 1).

The need for mental health support assessed here is limited to internalizing symptoms and suicidality, and the model was trained and validated on a sample enriched for these conditions. While this coverage addresses the most common clinical needs worldwide—depression and anxiety alone account for approximately 63% of mental health diagnoses [13]—the model’s performance on related (eg, eating disorders and posttraumatic stress disorder) or less related conditions (eg, attention-deficit/hyperactivity disorders and psychotic disorders) is unknown. Additionally, while the student and psychotherapist assessors also had access to participants’ descriptions of their social situation and support system, it is uncertain whether the LBA model captures, for example, social determinants (eg, loneliness) of support need. More broadly, applying the model to populations with different demographics, cultural backgrounds, or base rates of mental health conditions may reduce its accuracy or introduce biases [96]. This concern is consistent with prior work showing differential accuracy across racial or ethnic groups, such as regarding youth depression scores [97] and language markers of depression [98].

Lastly, this study assesses the need for mental health support on a 5-point scale, which has the advantage of being simple, interpretable, and comparable across individuals. Beyond the numerical output, the practical value of the scale lies in how the item formulations map onto a graded set of actions for those assessed. A complementary approach could involve using a generative LLM to produce personalized verbal support recommendations based on the individual’s responses. For example, lower scores could point to appropriate self-directed resources (eg, psychoeducation and lifestyle guidance), while higher scores could surface potentially suitable clinician-led initiatives and available crisis helplines. Embedding this into a digital health system could let the LBA function as a triage layer. The nondiagnostic, support-framed output may also help address attitudinal barriers to help-seeking by lowering reluctance and making screening feel lower-stakes. Future work could compare engagement with “need for support” vs diagnostic framings, and whether this effect is stronger among populations with higher baseline stigma around mental health conditions.

### Implications

The model could be used in research settings to screen participants’ need for mental health support, for example, to stratify study groups or identify individuals who may benefit from intervention. Its short application time and strong alignment with best-estimate assessments make it practical for large-scale screening where comprehensive clinical evaluation is not feasible.

In real-world settings, many people affected by mental health issues do not recognize a need for treatment, which makes help in early identification, support, and intervention crucial [2,4,7]. LBAs of the need for mental health support could complement existing screening tools and early intervention efforts. Possible applications include digital health platforms, informational websites on mood-related disorders, or health insurance portals, where individuals could receive preliminary guidance based on their responses. As the model assesses the need for support rather than psychiatric diagnoses, it may also help to reduce stigma surrounding mental health issues [33], encouraging more people to seek support. Implementing such a screening system in a real-world setting would require rigorous calibration to ensure fair, unbiased, and ethical prioritization. This is especially important when the target group differs in demographics, cultural background, or mental health condition base rates from the current enriched sample.

### Conclusions

This study demonstrates that an LBA model based on natural language responses can closely align with experienced psychotherapists’ assessments of need for mental health support, using less than 5 minutes of respondent time. The model demonstrated strong criterion, convergent, and face validity against best-estimate reference assessments based on longitudinal data. By assessing the overall need for support based on individuals’ own descriptions of their mental health, rather than predicting single diagnostic labels, this approach may offer a less stigmatizing and potentially a more flexible alternative suited for early-stage mental health screening. Both the comprehensive and parsimonious models are openly available at the LBA model library [54] to support further and independent validation.

## Supplementary material

10.2196/105460 Multimedia Appendix 1 Tables, figures, and exploratory analyses.

## Abbreviations

EU: European Union
GAD: Generalized Anxiety Disorder
GAD-7: Generalized Anxiety Disorder 7-Item Scale
HILS-3: Harmony in Life Scale 3
IDAS: Inventory of Depression and Anxiety Symptoms
LBA: language-based assessment
LDA: latent Dirichlet allocation
LEAD: Longitudinal Expert All Data
LIWC-22: Linguistic Inquiry and Word Count
LLM: large language model
MDD: major depressive disorder
PHQ-9: Patient Health Questionnaire-9
PSS-10: Perceived Stress Scale 10
SEMP: Sequential Evaluation With Model Preregistration
SWLS-3: Satisfaction With Life Scale 3
tf-idf: term frequency-inverse document frequency
