# Supplementary material for: Assessing the Need for Mental Health Support From Free-Text Responses: Development and Validation of Language-Based Assessments in Adults With Internalizing Symptoms
Source: JMIR Ment Health. 2026 Sep 25;13:e105460. doi: 10.2196/105460 (PMC13617639; doi:10.2196/105460)
Supplement: Supplementary file 1 [file mental-v13-e105460-s001.docx]

**Supplementary Materials - Additional Tables and Figures & Exploratory Analyses**

**Index**

[Additional Tables and Figures	3](#_Toc239598238)

[Methods	3](#_Toc239598239)

[Results	10](#_Toc239598240)

[Descriptive Statistics of Study Variables	10](#_Toc239598241)

[Optimizing Word Embeddings in Model Development	15](#_Toc239598246)

[Stepwise-Forward Procedure	17](#_Toc239598248)

[Descriptive Statistics of Language-Based Assessments	19](#_Toc239598249)

[Parsimonious Model	22](#_Toc239598250)

[Exploratory Analyses	24](#_Toc239598252)

[Comparison with Baseline Models	24](#_Toc239598253)

[Demographic Baseline	24](#_Toc239598254)

[TF-IDF Bag-of-Words	24](#_Toc239598255)

[Suicide Lexicon and LIWC	24](#_Toc239598256)

[Prognosis of Self-Reported Mental Health in 10 Weeks	28](#_Toc239598258)

[Comparing Model’s and Best-Estimate Topic Associations	30](#_Toc239598259)

[Convergence of Student and Best-Estimate Assessments	32](#_Toc239598261)

[Distribution-Based Correction of the LBAs	35](#_Toc239598263)

[References	37](#_Toc239598264)

# Additional Tables and Figures

## Methods

**Table S1**

*Exact Wording of the Text and Word Response Questions*

| Variable | Instruction |
| --- | --- |
| Mental health (t) | How is your mental health?  Please describe how you have been over the last two weeks. You can, for example, write about your emotions, thoughts, behaviors, and/or symptoms related to your health. Write at least one paragraph. |
| Social Situation (t) | Please describe the nature (or lack) of your close relationships. |
| Support System (t) | Please describe the nature (or lack) of your support system. |
| Medical History (t) | Describe your previous medical history; for example, when did you get sick, have you had similar or other problems before? |
| Self-Harm (t) | Please describe whether, and if so how, you have been intentionally harming yourself over the last two weeks. If you have not done this, please briefly describe that in your own words. |
| Suicidality (t) | Please describe whether, and if so how, you have been thinking about death, have had thoughts about killing yourself, have any intent or plan to kill yourself, or if you have attempted suicide over the last two weeks? If you have not had or done this, please briefly describe that in your own words. |
| Mental health (w) | Write or select five words that best describe your mental health over the last two weeks. Please choose all that apply: happy, positive, active, energetic, productive, hopeful, optimistic, strong, motivated, calm, sad, anxious, angry, hopeless, annoyed, numb, apathetic, alone, scared, irritated, other (specify). |
| Depression (w) | Write or select five words that best describe the level of depression that you have experienced, or not, over the last two weeks. Please choose all that apply: content, motivated, optimistic, happy, hopeful, fearful, energetic, lazy, okay, upbeat, suicidal, anxiety, angry, alone, numb, pessimistic, empty, lethargic, dejected, despondent, other (specify). |

*Note.* t = text response; w = word response.

**Table S2**

*Completed LEADING Guideline Reporting Checklist*

| Item | Name | Response |
| --- | --- | --- |
| **Longitudinal Design** | | |
| 1.1 | The time period | The participants were followed for ten weeks (N = 1404 at week 0 and N = 1228 at week 10), during which data relevant for the assessment of depression (Major Depressive Disorder; MDD), anxiety (Generalized Anxiety Disorder; GAD), stress (Stress Exhaustion Disorder; SED), suicidality, and self-harm were collected. |
| 1.2 | The number of time points | Data were collected through online surveys every second week (seven time points in total). The first and last assessment (week 0 and 10) consisted of a comprehensive mental health survey. The in-between assessments (week 2-8) consisted of shorter check-in surveys. |
| 1.3 | History or lifetime information | The assessments covered participants’ self-reported clinical history, including medical history, past mental/physical diagnoses, illnesses, treatments, sick leaves, healthcare visits, and family heredity. |
| 1.4 | The targeted time point(s) of the experts | The expert assessments targeted the first and last time points (week 0 and 10) and were based on data reports that included information from all time points. |
| **Appropriate Data** | | |
| 2.1 | The type and quality of the data | The comprehensive surveys consisted of open-ended questions, validated rating scales, and questions about clinical history and demographics. The check-in surveys covered a part of these open-ended questions and rating scales. A part of the sample (N = 212) participated in an unstructured clinical interview with a psychotherapist at the first time point.  The open-ended questions probed for text and word responses covering mental health, depression, anxiety, stress, self-harm, suicide, well-being, activities, reasons, life events, coping ability, medical history, social relationships, support system, and specific symptoms. The rating scales included he Patient Health Questionnaire-9 (PHQ-9); the Generalized Anxiety Disorder scale-7 (GAD-7); the Perceived Stress Scale-10 (PSS-10) for stress; the Inventory of Depression and Anxiety Symptoms (IDAS); the Harmony In Life Scale-3 (HILS-3); the Satisfaction With Life Scale-3 (SWLS-3); the WHO Disability Assessment Schedule (WHODAS); the Alcohol Use Disorders Identification Test (AUDIT); the Drug Use Disorders Identification Test (DUDIT); and the Self-reported DSM symptoms for depression and anxiety (SDAS), which is a self-report questionnaire reflecting a structured diagnostic interview. |
| 2.2 | The data triangulation | The data come from diverse methodological approaches, including open-ended questions, rating scales, self-reported behaviors, multiple- or single-choice questions, and an unstructured interview. |
| 2.3 | The data presentation | The raw data were presented to the experts as structured case reports in HTML format, where the experts could navigate across time points, data types, and constructs. |
| 2.4 | Access to the index measure | The experts had access to *all* collected raw data (including the raw language responses and the rating scale scores). |
| **Evaluation – experts, materials and procedures** | | |
| 3.1 | The expert and panel characteristics | The experts (2 women, 1 man; mean age 50.3 years) were psychotherapists (MSc - PhD) with more than five years of clinical experience in assessing and treating mental health issues. |
| 3.2 | The number of experts and panels | Three experts assessed the case reports. Regarding week 0, the participants who took part in the clinical interview (N = 212) received expert assessments from two to three experts, and another 167 participants received one expert assessment. |
| 3.3 | Blindness and conflicts of interest | The experts were blind to the research aims and had no conflicts of interest. |
| 3.4 | Instructions and training | The experts received written instructions part of the case reports, and the assessment sheet. Two experts took part in developing these instructions, providing feedback on the assessment sheet and its instructions as well as on the layout and presentation of the case reports. |
| 3.5 | The assessment procedure | MDD and GAD were assessed following DSM-5 criteria; SED was assessed following ICD-10 criteria. The experts first assessed the main symptoms of each disorder (e.g., low mood and loss of interest for MDD) before they decided on the diagnosis and severity of each disorder. After the three disorders, the experts assessed suicidality, self-harm, and the need for mental health support. |
| 3.6 | The assessment response format | The experts conducted their assessments for week 0 and week 10 in a structured digital assessment sheet. Scales were used to assess the severity of the main symptoms (1-7), the disorders (1-7), suicidality (0-3), self-harm (0-3), and the need for mental health support (1-5). Setting the diagnoses included three options (e.g., none/MDD/unspecified) as well as whether it was a primary or secondary diagnosis. |
| 3.7 | The data combination method | Experts had access to all survey data from all timepoints for their assessments for week 0 and week 10. No specific instructions for how to weigh and integrate the different data sources were provided, except to first assess the main symptoms before the disorder diagnosis and severity. |
| 3.8 | Independent expert assessments | All experts evaluated the data individually and made their individual assessments independently. |
| 3.9 | The inter-rater and inter-panel reliability | Krippendorff α was used to estimate the inter-rater reliability among the experts. |
| 3.10 | The solution to disagreements | The individual expert assessments were averaged into best-estimate assessments for the main symptoms, disorder severity, suicidality, self-harm, and the need for mental health support. Regarding diagnoses, the majority vote was taken (when two experts disagreed, it was made sure a third expert assessed the case independently). |
| **Validity** | | |
| 4.1 | The assessment description | The best-estimate assessments included MDD, GAD, and SED diagnoses as well as severity estimations of the disorders and their main symptoms, as well as for suicidality, self-harm, and the need for mental health support. |
| 4.2 | The validity and standard | The longitudinality, diversity of the data, and structured expert evaluations contribute to the validity of the best-estimate assessments. Limitations include the lack of a (semi-)structured interview and informant information as well as the experts having access *all* data (including language responses and rating scales) while it is uncertain how these sources were exactly weighted and integrated in their assessments. |

*Note.* This table was created according to the LEADING reporting guidelines [[1]](https://www.zotero.org/google-docs/?o18Hrx). In the current study, the participant data and best-estimate assessments from week 0 were used.

**Figure S1**

*Example Case Presented to the Graduate Student Assessors in the Online Study*

*Note.* The need for mental health support scale was presented in abbreviated form throughout the study. Participants could click on the blue box below to access the full descriptions of the scale points.

**Table S3**

*Completed GUIDE-LLM Reporting Checklist*

| Item | Name | Response |
| --- | --- | --- |
| **Scope of LLM use** | | |
| A.1 | LLMs were used in this project for | Generating contextual text embeddings from participants' language responses, which served as predictors in a ridge regression assessing need for mental health support (see *Methods - Model Configuration* and Figure 3). |
| A.2 | Degree of automation (human in the loop vs. fully automated) | Fully automated: embeddings were extracted through a scripted pipeline with no interactive LLM use; no LLM output required human review, as outputs were numerical embeddings rather than generated text. |
| **Model or system details** | | |
| B.1 | Model name, provider, size, version, date of access, source link | *mxbai-embed-large-v1* (Mixedbread AI); encoder-only transformer, 335 million parameters, 1,024-dimensional embeddings [[2]](https://www.zotero.org/google-docs/?XWx4Nu) |
| B.2 | Model access and context mode | Run locally via the *text* package (version 1.2.3) [[3]](https://www.zotero.org/google-docs/?pm00zI) in R, which retrieves the model from Hugging Face; no API or web interface. Each response was embedded in a separate call with no shared context across responses or participants. |
| B.3 | Relevant LLM configuration(s) reported, such as temperature, maximum tokens, seed and number of runs | Not applicable for sampling parameters (temperature, token limit, seed): the encoder produces deterministic embeddings and generates no text. Relevant embedding settings are reported in the *Methods* section: second-to-last layer, token averaging, concatenation across responses, and question prepending (see supplementary section *Optimizing Word Embeddings in Model Development*). |
| B.4 | Customization | None; the model was used off the shelf without fine-tuning. The only trained component was the downstream ridge regression (see *Methods - Model Configuration*). |
| B.5 | Did the LLM session(s) include persistent memory across interactions? | Not applicable; the encoder is stateless, and responses were embedded independently. |
| **Prompts** | | |
| C.1 | Exact prompts reported | The model received participants' responses with an abbreviated version of the corresponding survey question prepended. Exact question wordings are given in Table S1. |
| C.2 | System-wide instructions (if any) | Not applicable; embedding models take no system-level instructions. |
| **Data inputs and privacy** | | |
| D.1 | Handling of personal or sensitive data | Participants gave informed consent and an additional confirmation that their responses to suicide- and self-harm-related questions would not be acted upon (see *Methods - Ethical Considerations*). All embedding extraction was performed locally; no participant data were transmitted to third-party model providers. Free-text data are not publicly shared because they cannot be fully anonymized (Data Availability). |
| **Validation and interpretation** | | |
| E.1 | Human validation of LLM outputs | The language-based assessments were validated in a preregistered held-out set against best-estimate assessments from licensed psychotherapists (r = .82), against established clinical rating scales, and through face-validity analyses of linguistic topics (see *Methods - Model Validation* and *Results*). |
| E.2 | Relevant post-processing | Token embeddings were averaged per response and concatenated across language variables (Figure 3); model predictions outside the 1–5 scale range were truncated to 1 and 5 (*Methods - Model Configuration*). |
| **Reproducibility** | | |
| F.1 | Code, notebooks or scripts for LLM calls shared | The trained models are openly available in the L-BAM library [[4]](https://www.zotero.org/google-docs/?yHhzK6) and can be explored at the public demo page [[5]](https://www.zotero.org/google-docs/?QkhQ3E). Analysis code is available from the corresponding author upon reasonable request. |
| **Competing interests** | | |
| G.1 | Funding, support or other relevant relationship | Reported in the sections *Funding* and *Conflicts of Interest*; ONEK and KK have founded a start-up assessing mental health through AI-based language analysis. |

*Note.* This table was created according to the GUIDE-LLM reporting guidelines [[6]](https://www.zotero.org/google-docs/?4CV3xN).

## Results

### Descriptive Statistics of Study Variables

#### Natural Language Responses

Participants in the development set wrote between a median of 9 words (self-harm) and 40 words (mental health) in the text response variables. In the validation set, most mean and median word counts were slightly higher. See Table S4 for detailed descriptives of all language variables.

#### Rating Scales and Behavioral Measures

Participants' rating scale scores spanned the full or nearly full range, with good to excellent internal consistency (Cronbach's α = .85–.96; Table S5). A substantial proportion fell within clinically relevant ranges of at least moderate depression and/or anxiety (40.0% development, 44.3% validation [[7,8]](https://www.zotero.org/google-docs/?61cyNq))

#### Best-Estimate and Student Assessments

The assessed need for mental health support spanned the full range of 1 to 5 across both datasets and assessor groups. Participants in the development set received an average of 2.46 (SD = 1.12). In the validation set, participants received an average of 2.55 (SD = 1.07) from the students and 2.50 (SD = 1.16) from the psychotherapists; this difference was not significant, *t*(211) = 1.064, *p* = .289. The interrater reliability (Krippendorff’s α) among the students and the psychotherapists was .69 and .73, respectively. When comparing the mean assessment of the two assessor groups, the interrater reliability reached a satisfactory level (Krippendorff’s α = .82).

**Table S4**

*Descriptive Statistics of the Text and Word Responses.*

| Sample | Development Set (N = 600) | | | | Validation Set (N = 212) | | | | |
| --- | --- | --- | --- | --- | --- | --- | --- | --- | --- |
| Variable | MD | Mean | SD | Entropy | MD | Mean | SD | Entropy | |
| Mental health (t) | 40 | 50.5 | 30.8 | 8.8 | 53 | 64.3 | 43.4 | 8.6 |  |
| Social situation (t) | 30 | 38.9 | 29.1 | 8.3 | 35 | 45.7 | 29.6 | 8.1 | |
| Support system (t) | 27 | 31.9 | 17.9 | 8.2 | 29 | 36.2 | 20.8 | 8.1 | |
| Medical history (t) | 18 | 24.3 | 17.7 | 8.7 | 22 | 29.7 | 24.8 | 8.6 | |
| Self-Harm (t) | 9 | 12.6 | 11.6 | 7.3 | 9 | 12.8 | 11.8 | 7.1 | |
| Suicidality (t) | 12 | 17.8 | 18.7 | 7.8 | 12 | 19.9 | 22.8 | 7.6 | |
| Mental health (w) | 5 | 4.9 | 1.1 | 4.6 | 5 | 5.11 | 2.3 | 4.8 | |
| Depression (w) | 5 | 4.9 | 2.0 | 4.6 | 5 | 4.8 | 1.8 | 4.7 | |

*Note.* In the context of language, entropy quantifies both the diversity and unpredictability of word usage within each language variable. Higher entropy values indicate a greater variety of unique words and the presence of less predictable word choices, suggesting more informative and expressive language use. t = text response; w = word response. For all other variables, participants wrote a brief paragraph.

**Table S5**

*Descriptive Statistics of Rating Scales and Self-Reported Behavioral Measures*

| Scale | Development set (*N* = 600) | | | | | Validation set (*N* = 212) | | | | |
| --- | --- | --- | --- | --- | --- | --- | --- | --- | --- | --- |
|  | *M* | *SD* | *Md* | Range | $\alpha$ | *M* | *SD* | *Md* | Range | $\alpha$ |
| PHQ-9 | 8.13 | 6.50 | 7 | 0 – 27 | .90 | 8.66 | 6.94 | 7 | 0 – 26 | .91 |
| GAD-7 | 6.97 | 5.88 | 6 | 0 – 21 | .93 | 7.34 | 6.01 | 6 | 0 – 21 | .93 |
| PSS-10 | 17.23 | 8.84 | 17 | 0 – 38 | .91 | 18.37 | 9.08 | 19 | 0 – 40 | .92 |
| Suicidality | 8.20 | 3.87 | 6 | 6 – 30 | .86 | 7.99 | 3.52 | 6 | 6 – 29 | .85 |
| SWLS-3 | 12.17 | 5.10 | 13 | 3 – 21 | .93 | 11.24 | 5.28 | 11 | 3 – 21 | .94 |
| HILS-3 | 12.25 | 5.02 | 13 | 3 – 21 | .96 | 11.25 | 4.88 | 11 | 3 – 21 | .95 |
| Sick days | 1.69 | 11.19 | 0 | 0 – 92 | – | 2.99 | 13.67 | 0 | 0 – 92 | – |
| Healthcare visits | 0.27 | 1.42 | 0 | 0 – 19 | – | 0.54 | 2.96 | 0 | 0 – 38 | – |

*Note.* $\alpha$ = Cronbach’s alpha; PHQ-9 = Patient Health Questionnaire 9-item scale; GAD-7 = Generalized Anxiety Disorder 7-item scale; PSS-10 = Perceived Stress Scale 10-item version; Suicidality = Suicidality dimension of the Inventory of Depression and Anxiety Symptoms; SWLS-3 = Satisfaction With Life Scale 3-item version; HILS-3 = Harmony In Life Scale 3-item version; Sick days = number of sick days taken in the last 3 months due to mental health issues; Healthcare visits = number of healthcare visits taken in the last 3 months due to mental health issues.

**Table S6**

*Correlations of Study Variables in the Development Set (N = 600)*

| Variable | 1 | 2 | 3 | 4 | 5 | 6 | 7 | 8 | 9 | 10 |
| --- | --- | --- | --- | --- | --- | --- | --- | --- | --- | --- |
| 1. LBAs | 1 |  |  |  |  |  |  |  |  |  |
| 2. Student  assesssments | .87^***^ | 1 |  |  |  |  |  |  |  |  |
| 3. PHQ-9 | .78^***^ | .78^***^ | 1 |  |  |  |  |  |  |  |
| 4. GAD-7 | .74^***^ | .73^***^ | .82^***^ | 1 |  |  |  |  |  |  |
| 5. PSS-10 | .76^***^ | .74^***^ | .81^***^ | .83^***^ | 1 |  |  |  |  |  |
| 6. IDAS suicidality | .64^***^ | .66^***^ | .66^***^ | .56^***^ | .55^***^ | 1 |  |  |  |  |
| 7. SWLS-3 | -.65^***^ | -.64^***^ | -.65^***^ | -.59^***^ | -.68^***^ | -.45^***^ | 1 |  |  |  |
| 8. HILS-3 | -.70^***^ | -.69^***^ | -.71^***^ | -.66^***^ | -.74^***^ | -.46^***^ | .86^***^ | 1 |  |  |
| 9. Sick days | .22^***^ | .17^***^ | .23^***^ | .16^***^ | .18^***^ | .22^***^ | -.15^***^ | -.15^***^ | 1 |  |
| 10. Healthcare visits | .24^***^ | .24^***^ | .26^***^ | .21^***^ | .24^***^ | .28^***^ | -.15^***^ | -.17^***^ | .28^***^ | 1 |

*Note.* LBAs = Language-based assessments; PHQ-9 = Patient Health Questionnaire 9-item scale; GAD-7 = Generalized Anxiety Disorder 7-item scale; PSS-10 = Perceived Stress Scale 10-item version; IDAS Suicidality = Suicidality dimension of the Inventory of Depression and Anxiety Symptoms; Sick days = number of sick days taken in the last 3 months due to mental health issues; Healthcare visits = number of healthcare visits taken in the last 3 months due to mental health issues.
^*^ p < .05. ^**^ p < .01. ^***^ p < .001.

**Table S7**

*Correlations of Study Variables in the Validation Set (N = 212)*

| Variable | 1 | | 2 | | 3 | 4 | 5 | 6 | 7 | 8 | 9 | 10 |
| --- | --- | --- | --- | --- | --- | --- | --- | --- | --- | --- | --- | --- |
| 1. LBAs | 1 |  | |  | |  |  |  |  |  |  |  |
| 2. Best-estimate  assessments | .82^***^ | 1 | |  | |  |  |  |  |  |  |  |
| 3. Student  assessments | .87^***^ | .82^***^ | | 1 | |  |  |  |  |  |  |  |
| 4. PHQ-9 | .77^***^ | .84^***^ | | .75^***^ | | 1 |  |  |  |  |  |  |
| 5. GAD-7 | .72^***^ | .81^***^ | | .73^***^ | | .83^***^ | 1 |  |  |  |  |  |
| 6. PSS-10 | .76^***^ | .82^***^ | | .77^***^ | | .82^***^ | .83^***^ | 1 |  |  |  |  |
| 7. IDAS suicidality | .62^***^ | .61^***^ | | .62^***^ | | .63^***^ | .55^***^ | .51^***^ | 1 |  |  |  |
| 8. SWLS-3 | -.66^***^ | -.70^***^ | | -.66^***^ | | -.71^***^ | -.68^***^ | -.75^***^ | -.47^***^ | 1 |  |  |
| 9. HILS-3 | -.64^***^ | -.70^***^ | | -.65^***^ | | -.71^***^ | -.70^***^ | -.76^***^ | -.46^***^ | .85^***^ | 1 |  |
| 10. Sick days | .09 | .18^**^ | | .08 | | .13 | -07 | .14^*^ | .11 | -.10 | -.07 | 1 |
| 11. Healthcare visits | .16^*^ | .22^**^ | | .14^*^ | | .17^*^ | .14^*^ | .13 | .25^***^ | -.09 | -.04 | .19^**^ |

*Note.* LBAs = Language-based assessments; PHQ-9 = Patient Health Questionnaire 9-item scale; GAD-7 = Generalized Anxiety Disorder 7-item scale; PSS-10 = Perceived Stress Scale 10-item version; IDAS Suicidality = Suicidality dimension of the Inventory of Depression and Anxiety Symptoms; Sick days = number of sick days taken in the last 3 months due to mental health issues; Healthcare visits = number of healthcare visits taken in the last 3 months due to mental health issues.
 ^*^ p < .05. ^**^ p < .01. ^***^ p < .001.

### Optimizing Word Embeddings in Model Development

We explored three different methods of optimizing the word embeddings to increase model performance. To establish a preliminary variable set for the exploratory analysis, the stepwise-forward procedure was conducted using standard embeddings. Subsequently, the three optimization methods were tested on the same variable configuration. Each method’s effectiveness was evaluated by comparing the model residuals to those of the standard embeddings model using a paired *t*-test.

The first optimization method was averaging the embeddings across language variables. In the standard approach, embeddings from multiple variables were concatenated, increasing the number of dimensions in the ridge regression as variables were added (e.g., four variables result in a 4096-dimensional representation). Instead, this method averaged each embedding dimension across all language variables, maintaining a fixed 1024-dimensional input for the ridge regression, regardless of the number of variables.

The second method employed Matryoshka embeddings. Matryoshka representation learning allows models to retain most of their information even when dimensions are truncated [[8]](https://www.zotero.org/google-docs/?gV80kf). The *mxbai-large-v1* model was trained with a Matryoshka loss function, meaning that the most informative semantic features are stored in earlier embedding dimensions. By retaining only the first 512 of 1024 dimensions, this approach reduced the number of predictors in the ridge regression by half, potentially improving model efficiency.

In the third method, an abbreviated version of the question was prepended to each participant’s response before extracting the embedding. This approach provided additional contextual cues, allowing the model to incorporate the context of the response into its numerical representation. This potentially improves model performance by enhancing semantic encoding.

#### Results

The preliminary stepwise-forward procedure identified a three-variable LBA model using *mental health*, *suicidality*, and *medical history* paragraphs as input. This model already achieved a strong correlation between actual and predicted values of *r* = .849. Averaging the embeddings instead of concatenating them did not reduce model residuals (see Table S8 for significance tests) and led to a decline in model performance (*r* = .815). Similarly, using only the first 512 dimensions in Matryoshka embeddings did not reduce model residuals and slightly lowered model performance to *r* = .843. However, prepending the question prior to embedding extraction significantly reduced model residuals and increased model accuracy to *r* = .856. Consequently, the final stepwise-forward procedure was conducted using prepended question-embeddings, where an abbreviated version of each question was prepended to participant responses before embedding extraction.

**Table S8**

*Comparison of Exploratory LBA Models against a Preliminary LBA Model with Standard Embeddings*

| Embedding type | *r* | 95% CI | Mean abs. residual | $\Delta$ res | *t* | *df* | *p* |
| --- | --- | --- | --- | --- | --- | --- | --- |
| Standard embeddings | .849 | [.825, .870] | .459 |  |  |  |  |
| Averaged embeddings | .815 | [.787, .841] | .511 | -.052 | -4.882 | 599 | 1 |
| Matryoshka embeddings | .843 | [.818, .865] | .473 | -.014 | -3.672 | 599 | 1 |
| Prepended questions | .856 | [.833, .876] | .451 | .008 | 1.675 | 599 | .047 |

*Note.* LBA = Language-based assessment. All models used the paragraph variables *mental health*, *suicidality*, and *medical history* and were trained on the development set (*N* = 600). Each exploratory model was compared to the model with standard embeddings (first row) through a paired, one-sided *t*-test of model residuals. *r* = correlation between the actual and predicted values; Mean abs. residual = mean absolute residual of actual and predicted values; $\Delta$ res = mean difference of absolute model residuals.

### Stepwise-Forward Procedure

**Table S9**

*All Steps of the Stepwise-Forward Model Development Procedure*

| Baseline model | Additional variable | *r* | 95% CI | Mean absolute residual | $\Delta$ res | *t* | *df* | *p* |
| --- | --- | --- | --- | --- | --- | --- | --- | --- |
| Mental health (t) | – ^a^ | .789 | [.757, .817] | .547 |  |  |  |  |
|  | Social situation (t) | .803 | [.772, .829] | .528 | .019 | 2.096 | 599 | .018 |
|  | Support system (t) | .807 | [.777, .833] | .524 | .024 | 2.484 | 599 | .007 |
|  | Medical history (t) | .813 | [.784, .839] | .504 | .043 | 4.066 | 599 | < .001 |
|  | Self-harm (t) | .802 | [.772, .829] | .528 | .020 | 2.122 | 599 | .017 |
|  | Suicidality (t) ^b^ | .841 | [.816, .863] | .485 | .062 | 5.018 | 599 | < .001 |
|  | Mental health (w) | .807 | [.777, .833] | .517 | .031 | 2.765 | 599 | .003 |
|  | Depression (w) | .816 | [.787, .841] | .514 | .033 | 2.933 | 599 | .003 |
| mental health (t) +  suicidality (t) | – ^a^ | .841 | [.816, .863] | .485 |  |  |  |  |
|  | Social situation (t) | .846 | [.821, .867] | .475 | .011 | 1.428 | 599 | .077 |
|  | Support system (t) | .852 | [.829, .873] | .466 | .019 | 2.496 | 599 | .006 |
|  | Medical history (t) ^b^ | .856 | [.833, .876] | .451 | .034 | 3.841 | 599 | < .001 |
|  | Self-harm (t) | .838 | [.813, .861] | .490 | -.005 | -0.870 | 599 | .808 |
|  | General mental health (w) | .849 | [.826, .870] | .469 | .017 | 1.888 | 599 | .030 |
|  | Depression (w) | .849 | [.825, .870] | .472 | .014 | 1.632 | 599 | .052 |
| Mental health (t) +  suicidality (t) +  medical history (t) | – ^a^ | .856 | [.833, .876] | .451 |  |  |  |  |
|  | Social situation (t) | .862 | [.840, .881] | .447 | .004 | 0.574 | 599 | .283 |
|  | Support system (t) | .862 | [839., .881] | .448 | .002 | 0.383 | 599 | .351 |
|  | Self-harm (t) | .854 | [.830, .874] | .453 | -.002 | -0.342 | 599 | .634 |
|  | Mental health (w) | .862 | [.840, .881] | .442 | .008 | 1.030 | 599 | .152 |
|  | Depression (w) ^b^ | .865 | [.843, .884] | .438 | .013 | 1.661 | 599 | .049 |
| Mental health (t) +  suicidality (t) +  medical history (t) +  depression (w) | – ^a^ | .865 | [.843, .884] | .438 |  |  |  |  |
|  | Social situation (t) | .871 | [.850, .889] | .434 | .004 | .728 | 599 | .233 |
|  | Support system (t) | .870 | [.849, .888] | .435 | .003 | .630 | 599 | .265 |
|  | Self-harm (t) | .863 | [.841, .882] | .440 | -.002 | -.493 | 599 | .689 |
|  | General mental health (w) | .866 | [.841, .885] | .435 | .003 | .584 | 599 | .292 |

*Note.* This table shows all models tested within the stepwise-forward procedure. For each step (baseline model), the remaining variables were added one at a time. Paired, one-sided *t*-tests of model residuals statistically tested the added value of each variable. *r* = model accuracy (correlation between the actual and predicted values); $\Delta$ res = mean difference of absolute model residuals; t = text response; w = word response.

^a^ No additional variable (i.e., the baseline model).

^b^ Variable selected to add to the baseline model in this step.

### Descriptive Statistics of Language-Based Assessments

Participants in the development set received an average LBA of need for mental health support from the final model (see ‘Model Development’) of 2.47 (SD = 0.92), while those in the validation set had a similar average of 2.48 (SD = 0.89). While the best-estimate and student assessments rather followed a right-skewed uniform distribution, the LBAs approximated a normal distribution (Figure S2). A subsequent analysis of model residuals revealed a regression to the mean: the model tended to overestimate a low and underestimate a high need for support, as approximated by both the best-estimate and student assessments (Figure S3 and S4). Meanwhile, the mean of residuals was close to 0 for both datasets, indicating no general over- or underestimation across the sample (Figure S5).

**Figure S2**

*Distributions of Need for Mental Health Support Assessments*

**Figure S3**

*Residual Plots of the LBAs in Model Development and Validation*

*Note.* LBAs = Language-based assessments. Please note the different types of assessments on the x-axis. Residuals had a mean of -0.01 (*SD* = 0.56) in the development set and 0.02 (*SD* = 0.66) in the validation set. See Figure S4 for an example of an overestimated, correctly classified, and underestimated case, and Figure S5 for plots of predicted versus actual values and histograms of the residuals. The correlation between model residuals and human assessments was *r* _student_ = .55, *t*(598) = 16.28, *p* < .001 in model development and *r* _best-estimate_ = .64, *t*(210) = 12.15, *p* < .001 in model validation.

**Figure S4**

*Examples of an Overestimation, an Underestimation, and an Accurate LBA*

*Note.* LBA = language-based assessment; w = word response.

**Figure S5**

*Predicted versus Observed Need for Mental Health Support and Histograms of Residuals*

*Note.* Residuals had a mean of -0.01 (*SD* = 0.56) in the development set and 0.02 (*SD* = 0.66) in the validation set.

### Parsimonious Model

In addition to the final model, we explored a parsimonious model using only the mental health text variable. Such a parsimonious model offers practical advantages, as open questions about mental health are commonly included in studies and interviews, making it possible to use this model on existing datasets. Moreover, it requires less response time and computational time. We subjected the parsimonious model to the same validation analyses as in H1 to H3.

#### Results

The parsimonious model, using only *mental health* texts, demonstrated a strong performance only modestly lower than the full model. Its LBAs showed a correlation of *r* = .79 with the student assessments during model development, and a correlation of *r* = .77 with the best-estimate assessments in the validation set (H1; Table S10). Similar to the comprehensive model, it showed significant large correlations with the PHQ-9, GAD-7, PSS-10, SWLS-3, and HILS-3, but a comparatively small correlation with IDAS Suicidality scores (H2; Table S11). Correlations with the behavioral measures were in the expected direction, but weaker, and did not reach significance (H3; Table S11).

**Table S10**

*Comparison of the Parsimonious and Comprehensive LBA Model’s Performance*

| Performance measure | Parsimonious  LBA model | | Comprehensive LBA model | |
| --- | --- | --- | --- | --- |
|  | *r* | 95% CI | *r* | 95% CI |
| Training accuracy in the development set  (correlation with student assessments) | .789 | [.757, .817] | .865 | [.843, .884] |
| Criterion validity in the validation set  (correlation with best-estimate assessments) | .769 | [.708, .819] | .823 | [.774, .862] |
| Criterion validity in the validation set  with one-sided disattenuation  (correlation with best-estimate assessments) | .900 |  | .963 |  |

*Note.* LBA = Language-based assessment. The comprehensive LBA model is trained on the mental health text, suicidality text, medical history text, and depression word variables; the parsimonious LBA model is trained solely on the mental health texts. In the one-sided disattenuation, the correlation was corrected for the imperfect reliability of the psychotherapist assessments as estimated by Krippendorff’s $\alpha$ ($\alpha$ = .731).

**Table S11**

*Correlations of the Parsimonious Model’s LBAs with Rating Scales and Behavioral Measures in the Validation Set (N = 212) and Comparison to the Comprehensive Model*

| Variable | *r* _Parsimonious_ | 95% confidence interval | | *t* | *p* | *r* _Comprehensive_ |
| --- | --- | --- | --- | --- | --- | --- |
|  |  | lower limit | upper limit |  |  |  |
| PHQ-9 | 0.744 | 0.677 | 0.799 | 16.146 | <.001 | .767 |
| GAD-7 | 0.715 | 0.642 | 0.775 | 14.829 | <.001 | .718 |
| PSS-10 | 0.749 | 0.683 | 0.802 | 16.365 | <.001 | .761 |
| IDAS suicidality | 0.461 | 0.347 | 0.561 | 7.519 | <.001 | .620 |
| SWLS-3 | -0.631 | -0.706 | -0.542 | -11.789 | <.001 | -.663 |
| HILS-3 | -0.644 | -0.717 | -0.558 | -12.212 | <.001 | -.638 |
| Sick days | 0.056 | -0.079 | 0.190 | 0.816 | 0.415 | .086 |
| Healthcare visits | 0.109 | -0.026 | 0.240 | 1.589 | 0.114 | .157 |

*Note*. The comprehensive LBA model is trained on the mental health text, suicidality text, medical history text, and depression word variables; the parsimonious LBA model is trained solely on the mental health texts. PHQ-9 = Patient Health Questionnaire 9-item scale; GAD-7 = Generalized Anxiety Disorder 7-item scale; PSS-10 = Perceived Stress Scale 10-item version; IDAS Suicidality = Suicidality dimension of the Inventory of Depression and Anxiety Symptoms; Sick days = number of sick days taken in the last 3 months due to mental health issues; Healthcare visits = number of healthcare visits taken in the last 3 months due to mental health issues.

# Exploratory Analyses

## Comparison with Baseline Models

In an exploratory post-hoc analysis, we compared the LBAs against three baseline models to quantify the added value of contextual embeddings: a demographic model, a frequency-based bag-of-words model, and a lexicon-based model. The latter two models were not preregistered and were conducted in response to a reviewer. All baseline models were trained on the development set (*N* = 600) against the student assessments and evaluated on the held-out validation set (*N* = 212) against the best-estimate assessments, using the same ridge regression pipeline and the same 10-fold nested cross-validation as the LBA models. For each language-based baseline, we fitted a comprehensive variant using all four final model variables pasted together (*mental health*, *suicidality*, *medical history*, and *depression* words) and a parsimonious variant using only the *mental health* text responses, mirroring the two LBA models.

### Demographic Baseline

The demographic model used age, gender, and occupational status as predictors; the same information the graduate student raters saw during the annotation process. In addition to the baseline model, we added this demographic information to the final embedding model to investigate if adding demographic information would significantly improve it.

### TF-IDF Bag-of-Words

The text responses were lowercased, stripped of punctuation and numbers, and stemmed using the Snowball stemmer. We removed stopwords using the Snowball stopword list, but we retained all negators (e.g., "not", "never", "no", "don't"), since negation is central to the responses in the *Suicide* variable (see Figure 5). Documents were tokenized into 1- to 3-grams and weighted by term frequency–inverse document frequency (TF-IDF). Terms occurring in fewer than 3% of documents were removed. The *depression* word responses were processed as a separate unigram document-term matrix and concatenated to the n-gram features to prevent the formation of nonsensical n-grams from the response words. For the validation set, features were projected onto the development vocabulary using the inverse document frequencies estimated in the development set, so that no information from the held-out set entered feature construction.

### Suicide Lexicon and LIWC

This model combined two lexicon-based feature sets. First, we developed a suicide-term dictionary with negation detection: multi-word suicide- and self-harm-related terms were matched in each response, and each match was classified as affirmed or negated depending on whether a negator preceded it within the same clause. Clause-level rather than fixed-window scope was used because common phrasings span five or more tokens (e.g., "I have not had any thoughts about killing myself"). Affirmed and negated mention rates per 100 words were entered as separate features. Second, we computed all LIWC-22 dimensions [[9]](https://www.zotero.org/google-docs/?0bgvi9) for each language variable, excluding only the segment identifier and dimensions with zero variance. We did not preselect LIWC dimensions to not advantage the baseline through ad-hoc selection.

### Results

The LBAs outperformed all baseline models in both the development and validation sets (Figure S6; Table S12). In the held-out validation set, the comprehensive LBA achieved a criterion validity against the best-estimate assessments of *r* = .82, compared with *r* = .75 for the TF-IDF model and *r* = .69 for the lexicon-based model; the demographic baseline reached only *r* = .22. The difference between the comprehensive LBA and each baseline was statistically significant [paired one-sided *t*-tests of absolute residuals: *t*(211) = 5.06, *p* < .001 vs. TF-IDF; *t*(211) = 5.61, *p* < .001 vs. lexicon].

The magnitude of the LBAs' advantage depended strongly on the amount of available text. For the comprehensive models drawing on four language responses, the advantage over the baseline language models was moderate (Δ*r* = .07 for TF-IDF and Δ*r* = .13 for suicide lexicon and LIWC, see Figure S6). For the parsimonious models based on a single *mental health* response, it was substantial (Δ*r* = .28 for TF-IDF and Δ*r* = .21 for suicide lexicon and LIWC). Frequency- and dictionary-based representations require a sufficient number of matching terms per document, which short responses (median = 53 words in the validation set) often do not provide. On the contrary, contextual embeddings represent brief responses as dense semantic vectors, facilitating accurate predictions.

Notably, the atheoretical TF-IDF model outperformed the curated lexicon-based model in both sets, indicating that a data-driven vocabulary captures more relevant variance than predefined dictionaries even when these are tailored to the assessment domain. All language-based models substantially outperformed the demographic baseline, confirming that the assessments are driven by the language responses rather than by demographic characteristics. Adding the demographic variables to the final embedding model did not provide any additional predictive value beyond the language variables, *t*(599) = 1.153, *p* = .476, yielding a nearly identical accuracy of *r* = .866. These comparisons should be interpreted as exploratory. We further note that the suicide lexicon and LIWC model rely on the proprietary LIWC-22 dictionary, whereas Model A can be reproduced with open-source tools.

**Figure S6**

*Accuracy of the LBAs and Baseline Models in the Development and Validation Set.*

*Note. Error bars represent 95% confidence intervals.*

**Table S12**

*Test of Baseline Model Residuals against Embedding Models*

| Baseline model | Mean abs. residual | Mean abs. residual of embedding model | Mean diff. | *t* | *df* | *p* |
| --- | --- | --- | --- | --- | --- | --- |
| **Development (compared against student assessments)** | | | | | | |
| TF-IDF | 0.560 | 0.438 | 0.122 | 8.000 | 599 | <.001 |
| Suicide lexicon and LIWC | 0.641 | 0.438 | 0.203 | 11.168 | 599 | <.001 |
| TF-IDF - parsimonious | 0.748 | 0.547 | 0.201 | 9.785 | 599 | <.001 |
| Suicide lexicon and LIWC - parsimonious | 0.754 | 0.547 | 0.206 | 10.112 | 582 | <.001 |
| **Validation (compared against best-estimate assessments)** | | | | | | |
| TF-IDF | 0.668 | 0.536 | 0.132 | 5.055 | 211 | <.001 |
| Suicide lexicon and LIWC | 0.712 | 0.536 | 0.176 | 5.609 | 211 | <.001 |
| TF-IDF - parsimonious | 0.862 | 0.588 | 0.274 | 7.875 | 211 | <.001 |
| Suicide lexicon and LIWC - parsimonious | 0.802 | 0.583 | 0.219 | 6.403 | 204 | <.001 |

*Note*. Paired one-sided t-test on absolute model residuals. TF-IDF = Term frequency-inverse document frequency; LIWC = Linguistic Inquiry and Word Count.

## Prognosis of Self-Reported Mental Health in 10 Weeks

In an exploratory post-hoc analysis, we tested whether the model's LBA carries prognostic information. We combined the development and validation sets and predicted participants' scores on four clinical scales measured 10 weeks later (T2). These were the same scales used for model validation: depression (PHQ-9), anxiety (GAD-7), perceived stress (PSS-10), and suicidality (IDAS-Suicidality).

We were able to combine both sets for this analysis because the clinical scales were never used to train the model. It was trained solely on the graduate students’ ratings, who only saw the participants’ language responses and demographic information. Therefore, predicting the T2 scales was an out-of-sample task across the entire pooled sample (n = 733, dropout = 9.7%), which increased statistical power.

We asked whether the LBA at T1 predicts a scale at T2 beyond that scale's own value at T1. For each scale, we fit a multiple linear regression with the T2 score as the dependent variable. The predictors were the T1 score of the same scale and the T1 LBA.

As expected for relatively stable clinical constructs, the T1 score was the strongest predictor in every model. Beyond it, the T1 LBA was a significant predictor of every scale at T2 (all *p* ≤ .002; see Table S13). Higher LBA scores predicted higher symptom levels 10 weeks later, and this held consistently across depression, anxiety, stress, and suicidality.

The additional variance was small. The LBA explained between 0.5% and 1.2% of variance beyond the T1 scale (Δ*R²* = .005–.012). This was expected, as the T1 scale already accounts for most of the reliable variance in the T2 scale. Overall, the LBA holds a small but consistent amount of prognostic information about future self-reported symptoms, over and above the clinical scale itself. Given the exploratory design, these results should be read as preliminary.

**Table S13**

*Predictive prognosis power of the Model’s LBAs 10 weeks later (T2) - beyond the scale itself*

| Outcome / Predictor | *b* | Std. error | *t* | *p* |
| --- | --- | --- | --- | --- |
| **PHQ-9 (T2)** |  |  |  |  |
| Intercept | 0.203 | 0.500 | 0.410 | 0.682 |
| LBA (T1) | 0.814 | 0.271 | 3.005 | 0.003 |
| PHQ-9 (T1) | 0.610 | 0.032 | 16.405 | < 0.001 |
| *R*^2^ = .556, Δ*R*^2^_LBA_ = .006, *n* = 733 | | | | |
| **GAD-7 (T2)** |  |  |  |  |
| Intercept | -0.382 | 0.457 | -0.835 | 0.401 |
| LBA (T1) | 1.042 | 0.237 | 4.398 | < 0.001 |
| GAD-7 (T1) | 0.559 | 0.037 | 15.310 | < 0.001 |
| *R*^2^ = .513, Δ*R*^2^_LBA_ = .013, *n* = 733 | | | | |
| **PSS-10 (T2)** |  |  |  |  |
| Intercept | 2.234 | 0.673 | 3.320 | < 0.001 |
| LBA (T1) | 1.740 | 0.396 | 4.396 | < 0.001 |
| PSS-10 (T1) | 0.564 | 0.040 | 14.006 | < 0.001 |
| *R*^2^ = .506, Δ*R*^2^_LBA_ = 013, *n* = 733 | | | | |
| **IDAS-Suicidality (T2)** |  |  |  |  |
| Intercept | 1.440 | 0.268 | 5.362 | < 0.001 |
| LBA (T1) | 0.492 | 0.129 | 3.799 | < 0.001 |
| IDAS-Suicidality (T1) | 0.648 | 0.032 | 20.464 | < 0.001 |
| *R*^2^ = .551, Δ*R*^2^_LBA_ = .009, *n* = 733 | | | | |

*Note*. LBA = Language-based assessments; PHQ-9 = Patient Health Questionnaire 9-item scale; GAD-7 = Generalized Anxiety Disorder 7-item scale; PSS-10 = Perceived Stress Scale 10-item version; IDAS Suicidality = Suicidality dimension of the Inventory of Depression and Anxiety Symptoms.

## Comparing Model’s and Best-Estimate Topic Associations

To investigate potential differences in how the model versus the psychotherapists assess the need for mental health support, we compared the correlations between LDA topics and both the LBAs and best-estimate assessments. We used the same LDA topics extracted from the full sample (*N* = 812) but tested the correlations only within the validation set (*N* = 212), as the best-estimate assessments were only available for this subset. False Discovery Rate corrections were applied within each variable. We visualized the topic correlations in a two-dimensional plot to identify potential differences in the language patterns associated with each level of need for mental health support.

### Results

Correlations between LDA topics and the LBAs (x-axis; see Figure S7) closely aligned with those between LDA topics and the best-estimate assessments (y-axis). The correlations clustered around the angle bisector, indicating that both the LBAs and best-estimate assessments correlated with linguistic topics in a similar way. While the topics’ correlation sizes were comparable to those in the full sample, fewer topics reached statistical significance, likely because of the smaller sample size when using only the validation set.

**Figure S7**

*Correlations of LDA topics with the LBAs (x-axis) and Best-Estimate Assessments (y-axis)*

*Note.* LBAs = Language-Based Assessments. Both the x- and y-axes represent the magnitude of correlation coefficients. The topics were derived from the full sample (*N* = 812), while correlations were calculated and tested using only the validation sample (*N* = 212). False Discovery Rate (FDR) Type-I error corrections were applied within each variable.

## Convergence of Student and Best-Estimate Assessments

In a post-hoc exploratory analysis, we examined where the student and best-estimate assessments converged and diverged in the validation set (*N* = 212). While the two assessor groups showed similar averages and high agreement (Krippendorff's α = .82), the students based their assessments solely on baseline language responses and basic demographics, whereas the psychotherapists had access to all longitudinal data, including rating scale scores. We therefore tested whether the psychotherapists' divergence from the students was unsystematic or traceable to information only the psychotherapists had seen (i.e., the clinical rating scales). Using hierarchical linear regressions, we predicted the best-estimate assessments from the averaged student assessments (Model 1), added the four clinical rating scales as a block (PHQ-9, GAD-7, PSS-10, IDAS Suicidality; Model 2), and finally added the LBAs (Model 3). For simplicity, this analysis disregards the further information available to the psychotherapists (e.g., longitudinal trajectories, clinical interview, sociodemographics, and clinical history), for which the baseline scales serve as a partial proxy.

### Results

A Tukey mean difference plot (Figure S8) showed close convergence: the mean difference between the averaged student and best-estimate assessments was 0.05 (*SD* = 0.68), and only 20 of 212 cases (9%) diverged by more than one scale point on the need for mental health support scale. The difference showed a weak but non-significant negative association with assessed severity (*r* = -.13, *p* = .053). The numeric result indicates that the psychotherapists assigned relatively higher values to high-severity cases and used the scale range somewhat more fully than the students (which is also reflected in the larger *SD* of their ratings - *SD* = 1.16 vs. 1.07 for the students).

The student assessments alone explained 67% of the variance in the best-estimate assessments (Model 1; Table S14). Adding the rating scales significantly increased the explained variance (Δ*R²* = .14, *F*(4, 206) = 39.00, *p* < .001), indicating that the divergence between the assessor groups was largely systematic and aligned with the questionnaire information that only the psychotherapists had seen. Within the scales, depression (PHQ-9), anxiety (GAD-7), and stress scores (PSS-10) contributed significantly, whereas IDAS Suicidality did not - plausibly because suicidality was explicitly probed in the language responses available to the students, and because the scale showed a strong floor effect in this sample (median = 6, the scale minimum; see Table S5).

Adding the LBAs further improved the model (Δ*R²* = .009, *F*(1, 205) = 9.70, *p* = .002), suggesting that the model extracts language-based severity signals beyond what the student assessors captured. Notably, the final model explained 82% of the variance in the best-estimate assessments. Given the interrater agreement among psychotherapists (*r* = .78), the reliable variance of their averaged assessments is approximately 85–90% (Spearman–Brown). The combination of student assessments, rating scales, and LBAs thus reconstructs nearly all of the reliable variance in the expert assessments. Importantly, this analysis cannot establish that the psychotherapists directly *used* the scale scores, as the scales may correlate with severity information the psychotherapists inferred from other sources.

**Figure S8**

*Tukey Mean Difference Plot of Average Student and Best-Estimate (Psychotherapist) Recommendations in the Validation Set*

*Note.* Each dot represents a participant in the validation set (*N* = 212). The dots are slightly jittered to improve readability. The blue line represents the mean difference between the recommendations of the two assessor groups (*M* = 0.05, *SD* = 0.68), while the red lines indicate the 95% confidence interval of this difference. Positive y-values indicate that the student raters gave a higher need for mental health support rating, while negative y-values indicate that the psychotherapists gave a higher rating.

**Table S14**

*Hierarchical Linear Regressions Predicting the Best-Estimate Assessments in the Validation Set (N = 212)*

| Outcome / Predictor | *b* | Std. error | *t* | *p* |
| --- | --- | --- | --- | --- |
| **Model 1** |  |  |  |  |
| Intercept | 0.250 | 0.119 | 2.101 | 0.037 |
| Student assessment | 0.883 | 0.043 | 20.547 | < 0.001 |
| *R*^2^ = 0.668 | | | | |
| **Model 2** |  |  |  |  |
| Intercept | 0.452 | 0.116 | 3.894 | < 0.001 |
| Student assessment | 0.326 | 0.058 | 5.647 | < 0.001 |
| PHQ-9 | 0.049 | 0.011 | 4.525 | < 0.001 |
| GAD-7 | 0.033 | 0.012 | 2.755 | 0.006 |
| PSS-10 | 0.023 | 0.008 | 2.891 | 0.004 |
| IDAS-Suicidality | 0.016 | 0.014 | 1.185 | 0.237 |
| *R*^2^ = 0.811, Δ*R*^2^_Model 1_ = 0.143, *F*(4, 206) = 39.00, *p* < .001 | | | | |
| **Model 3** |  |  |  |  |
| Intercept | 0.298 | 0.124 | 2.406 | 0.017 |
| Student assessment | 0.192 | 0.071 | 2.708 | 0.007 |
| PHQ-9 | 0.043 | 0.012 | 4.082 | < 0.001 |
| GAD-7 | 0.033 | 0.012 | 2.831 | 0.005 |
| PSS-10 | 0.020 | 0.008 | 2.536 | 0.012 |
| IDAS-Suicidality | 0.011 | 0.013 | 0.850 | 0.396 |
| LBA | 0.258 | 0.083 | 3.115 | 0.002 |
| *R*^2^ = 0.820, Δ*R*^2^_Model 2_ = 0.009, *F*(1, 205) = 9.70, *p* = 0.002 | | | | |

*Note*. PHQ-9 = Patient Health Questionnaire 9-item scale; GAD-7 = Generalized Anxiety Disorder 7-item scale; PSS-10 = Perceived Stress Scale 10-item version; IDAS Suicidality = Suicidality dimension of the Inventory of Depression and Anxiety Symptoms; LBA = Language-based assessments.

## Distribution-Based Correction of the LBAs

Because ridge regularization shrinks predictions toward the criterion mean, the LBAs showed a regression-to-the-mean effect (Figures S2–S5). In an exploratory post-hoc analysis, we demonstrated two distribution-based corrections that are readily applicable without retraining the model: (1) linear variance matching, which rescales the predictions to the mean and standard deviation of the human assessments, and (2) empirical quantile mapping, which maps the predictions onto the full distribution of the human assessments. Both corrections were estimated solely in the development set (i.e., predictions were reshaped to match the student assessments) and applied unchanged to the held-out validation set, where they were evaluated against the best-estimate assessments. Corrected values were truncated to the 1–5 scale range.

Both corrections left criterion validity essentially unchanged (*r* = .82-.83 with the best-estimate assessments) while substantially improving calibration (Figure S9, Table S15): First, the spread of the predictions approached that of the best-estimate assessments (*SD* = 0.89 uncorrected vs. 1.04 and 1.10 corrected; best-estimates: 1.16). (2) Second, the correlation between residuals and assessed severity decreased from .64 to .45 (linear) and .38 (quantile). Third, the systematic overestimation of cases with low need for support was reduced by 29-40%, and the systematic underestimation of cases with high need for support was reduced by 44-45%, as indicated by the mean residuals for cases with best-estimate assessments ≤ 2 and ≥ 4, respectively; all without increasing the mean absolute residuals. As the corrections re-map predictions rather than improve the model's ranking accuracy, they trade a small amount of aggregate precision (RMSE) for calibration at the distributional extremes, where, especially at the high end, assessment decisions are most consequential.

**Figure S9**

*Distributions of LBAs with different Distribution-Based Corrections*

*Note.* LBAs = Language-based assessments.

**Table S15**

*Evaluation of Two Distribution-Based Corrections of the LBAs Against the Best-Estimate Assessments in the Validation Set (N = 212)*

| Model | *r* | *SD* of LBAs | *r*_resid best-estimate_ | Mean abs. residual | RMSE | Mean res low | Mean res high |
| --- | --- | --- | --- | --- | --- | --- | --- |
| Uncorrected | 0.82 | 0.89 | 0.64 | 0.526 | 0.658 | -0.371 | 0.613 |
| Linear variance matching | 0.83 | 1.04 | 0.45 | 0.513 | 0.657 | -0.265 | 0.399 |
| Quantile mapping | 0.82 | 1.10 | 0.38 | 0.519 | 0.681 | -0.223 | 0.343 |

*Note.* LBA = language-based assessment; *r* = Pearson correlation between the (corrected) LBAs and the best-estimate assessments; *r*_resid best-estimate_ = correlation between residuals and best-estimate assessments; Mean abs. residual = mean absolute residual; RMSE = root mean squared error; Mean res low = mean residual among cases with low need for support (best-estimate ≤ 2), with negative values indicating overestimation; Mean res high = mean residual among cases with high need for support (best-estimate ≥ 4), with positive values indicating underestimation.

# References

[1.](https://www.zotero.org/google-docs/?sxjoqZ)	[Eijsbroek VC, Kjell K, Schwartz HA, Boehnke JR, Fried EI, Klein DN, Gustafsson P, Augenstein I, Bossuyt PMM, Kjell ONE. The LEADING guideline: Reporting standards for expert panel, best-estimate diagnosis, and longitudinal expert all data (LEAD) methods. Compr Psychiatry 2025 Aug 1;141:152603. doi: 10.1016/j.comppsych.2025.152603](https://www.zotero.org/google-docs/?sxjoqZ)

[2.](https://www.zotero.org/google-docs/?sxjoqZ)	[Li X, Li J. AnglE-optimized Text Embeddings. arXiv; 2024. Available from: http://arxiv.org/abs/2309.12871 [accessed Sept 16, 2024]](https://www.zotero.org/google-docs/?sxjoqZ)

[3.](https://www.zotero.org/google-docs/?sxjoqZ)	[Kjell O, Giorgi S, Schwartz HA. The Text-Package : An R-Package for Analyzing and Visualizing Human Language Using Natural Language Processing and Transformers. Psychol Methods 2023 Jan 1;28(6):1478–1498. doi: 10.1037/met0000542](https://www.zotero.org/google-docs/?sxjoqZ)

[4.](https://www.zotero.org/google-docs/?sxjoqZ)	[Nilsson AH, Eijsbroek VC, Gu Z, Kjell K, Giorgi S, Kotov R, Ganesan AV, Schwartz HA, Kjell ONE. The Language-Based Assessment Model Library: Open Model Sharing for Independent Validation and Broader Applications. Adv Methods Pract Psychol Sci SAGE Publications Inc; 2026 Apr 1;9(2):25152459261419036. doi: 10.1177/25152459261419036](https://www.zotero.org/google-docs/?sxjoqZ)

[5.](https://www.zotero.org/google-docs/?sxjoqZ)	[Mental Health Recommendations - a Hugging Face Space by cwiebel. Available from: https://huggingface.co/spaces/cwiebel/mental-health-recommendations [accessed June 24, 2026]](https://www.zotero.org/google-docs/?sxjoqZ)

[6.](https://www.zotero.org/google-docs/?sxjoqZ)	[Feuerriegel S, Barrie C, Crockett MJ, Globig LK, McLoughlin KL, Mirea D-M, Spirling A, Yang D, Althoff T, Antoniak M, Argyle LP, Ashokkumar A, Atari M, Bailey H, Bauer K, Bhatt U, Chai Y, Chakraborty T, Chandra Y, Chen H, Daumé H, De Francisci Morales G, Dehghani M, Dillion D, Eichstaedt JC, Forster K, Geissler D, Gray K, Griffiths TL, Jochen H, Hauser OP, He JK, Hemrajani R, Holzmeister F, Hwang AH-C, Hu T, Ivanova AA, Köbis N, Kyrychenko Y, Lakkaraju H, Liu J, Maarouf A, Maier S, Meincke L, Mihalcea R, Mittelstadt B, Mohammad SM, Naaman M, Netzer O, Oh A, Ong DC, Pierri F, Plank B, Rahwan I, Rahwan T, Rao PSB, Robertson CE, Rothschild DM, Salganik MJ, Schulz E, Shah C, Shrestha YR, Shutova E, Siegel AA, Simchon A, Sun H, Toetzke M, Van Bavel JJ, Vaccaro M, Wortman Vaughan J, Vayena E, Vaz-de-Melo POS, Vecchione B, Wang A, West R, Willer R, Wulff DU, Zhang R, Zhang S, Rathje S, Horta Ribeiro M. A reporting checklist for large language models in behavioural science. Nat Hum Behav Nature Publishing Group; 2026 July;10(7):1182–1186. doi: 10.1038/s41562-026-02492-7](https://www.zotero.org/google-docs/?sxjoqZ)

[7.](https://www.zotero.org/google-docs/?sxjoqZ)	[Kroenke K, Spitzer RL, Williams JBW. The PHQ-9: Validity of a brief depression severity measure. J Gen Intern Med 2001 Sept;16(9):606–613. doi: 10.1046/j.1525-1497.2001.016009606.x](https://www.zotero.org/google-docs/?sxjoqZ)

[8.](https://www.zotero.org/google-docs/?sxjoqZ)	[Kusupati A, Bhatt G, Rege A, Wallingford M, Sinha A, Ramanujan V, Howard-Snyder W, Chen K, Kakade S, Jain P, Farhadi A. Matryoshka Representation Learning. arXiv; 2024. doi: 10.48550/arXiv.2205.13147](https://www.zotero.org/google-docs/?sxjoqZ)

[9.](https://www.zotero.org/google-docs/?sxjoqZ)	[Boyd RL, Ashokkumar A, Seraj S, Pennebaker JW. The development and psychometric properties of LIWC-22. Austin, Texas: University of Texas at Austin; 2022. Available from: https://www.liwc.app](https://www.zotero.org/google-docs/?sxjoqZ)
